# HLA I immunopeptidome of synthetic long peptide pulsed human dendritic cells for therapeutic vaccine design

**DOI:** 10.1038/s41541-025-01069-1

**Published:** 2025-01-18

**Authors:** Amy L. Kessler, Roel F. A. Pieterman, Wouter A. S. Doff, Karel Bezstarosti, Rachid Bouzid, Kim Klarenaar, Diahann T. S. L. Jansen, Robbie J. Luijten, Jeroen A. A. Demmers, Sonja I. Buschow

**Affiliations:** 1 Department of Gastroenterology & Hepatology, Erasmus MC University Medical Center Rotterdam, Rotterdam, The Netherlands; 2 Proteomics Center, Department of Biochemistry, Erasmus MC University Medical Center Rotterdam, Rotterdam, The Netherlands; 3 Present Address: Biomolecular Mass Spectrometry and Proteomics, Bijvoet Center for Biomolecular Research and Utrecht Institute for Pharmaceutical sciences, University of Utrecht, Utrecht, The Netherlands; 4 Present Address: Merus N.V., Utrecht, The Netherlands; 5 Present Address: Division of Laboratories, Pharmacy and Biomedical Genetics, UMC Utrecht, Utrecht, The Netherlands; 6 Present Address: Department of Internal Medicine, Leiden University Medical Center, Leiden, The Netherlands

**Keywords:** Antigen presentation, Peptide vaccines, Hepatitis B

## Abstract

Synthetic long peptides (SLPs) are a promising vaccine modality that exploit dendritic cells (DC) to treat chronic infections or cancer. Currently, the design of SLPs relies on in silico prediction and multifactorial T cells assays to determine which SLPs are best cross-presented on DC human leukocyte antigen class I (HLA-I). Furthermore, it is unknown how TLR ligand-based adjuvants affect DC cross-presentation. Here, we generated a unique, high-quality immunopeptidome dataset of human DCs pulsed with 12 hepatitis B virus (HBV)-based SLPs combined with either a TLR1/2 (Amplivant®) or TLR3 (PolyI:C) ligand. The obtained immunopeptidome reflected adjuvant-induced differences, but no differences in cross-presentation of SLPs. We uncovered dominant (cross-)presentation on B-alleles, and identified 33 unique SLP-derived HLA-I peptides, several of which were not in silico predicted and some were consistently found across donors. Our work puts forward DC immunopeptidomics as a valuable tool for therapeutic vaccine design.

## Introduction

Immunotherapy aims to reinvigorate the patient’s adaptive immunity by prompting cytotoxic CD8 + T lymphocytes (CTLs) to recognize and attack aberrant cells that present immunogenic foreign peptides (i.e. *epitopes*) on human leukocyte antigen class I (HLA-I) molecules^1^. Antigen-based immunotherapies are designed to exploit dendritic cells (DCs) to specifically instruct the patient’s T cells to recognize tumor- or pathogen-specific epitopes on diseased target cells. Subsequently, and when trained by an appropriately activated DC, these antigen-specific T cells can then eradicate the diseased target cell. Within the field of antigen-based therapies, therapeutic vaccination has become increasingly popular due to its ability to enhance pre-existing T cell immunity as well as induce de novo T cell responses^2^. Alongside checkpoint inhibitors and other therapies to lift ongoing immune suppression, therapeutic vaccination has already shown promising clinical results^3–6^.

Highly promising vaccine modalities are synthetic long peptides (SLPs) and the more recently emerging mRNA vaccines^7–9^. While mRNA vaccines may be most straightforward to produce, peptides are more stable even at ambient temperatures, facilitating use of SLP vaccines also in low-income countries where freezer facilities may be more limited. In addition, peptides do not rely on translation and hence are one step closer to HLA presentation and give more control over the amount of antigen offered.

SLPs are 25–40 amino acid (AA) long peptide stretches based on the sequence of tumor or viral antigens^8^. Unlike single epitope short peptides, which can be presented by any cell and thereby induce tolerance, SLPs are selectively taken up by antigen presenting cells (APCs), such as DCs, for processing and epitope presentation to T cells^9,10^. Furthermore, SLPs may contain various HLA-I and HLA class II (HLA-II) epitopes, creating a concentrated yet broad T cell priming potential^11–13^. Multiple SLPs can be combined to create a generic vaccine effective for each patient irrespective of their HLA type or viral genotype. So far, the SLP concept has shown encouraging clinical efficacy for HPV (-induced malignancies) as well as other solid tumors^4,14–16^. The design of an effective SLP-based vaccine, however, is complex.

The efficacy of SLP-based therapeutic vaccination heavily relies on APCs to prime T cell responses. DCs have an exceptional ability to present exogenous antigens, such as SLPs, with high efficiency on HLA-I (i.e. *cross-presentation*) to prime CTLs. T cell priming consists of the recognition of a DC-presented HLA-bound peptide by the T cell receptor (TCR)^17^. To induce clinically relevant CD8 + CTL responses, it is crucial that the DC not only presents HLA peptides that are also naturally presented on by the target cell^18^, but also expresses co-stimulatory molecules and cytokines to further promote CTL expansion, longevity and effector function. This CTL-promoting phenotype can results from either interaction with activated CD4 + T helper cells^19^, or stimulation with adjuvants such as Toll-like receptor (TLR) ligands^20^. Hence, by supplementing SLP vaccines with adjuvants based on TLR ligands a mature DC phenotype is induced that is optimal for T cell priming^21,22^. Signal transduction via TLRs commonly triggers DC maturation and cytokine production via MyD88 and downstream TRAF6 signaling^23^. TLR3, however, relies on TRIF signaling that, besides TRAF6, also propagates signals via TRAF3, inducing an alternative phenotype of mature DCs skewed to eliminating viruses by secreting more type I interferons^23,24^. Therefore, the choice of vaccine adjuvant can determine the direction of the immune response^25,26^. It is currently unknown, however, whether the (cross-)presented repertoire of HLA peptides (i.e., *immunopeptidome*) differs between DCs matured with distinct TLR ligands signaling via MyD88 or TRIF^27^.

Chronic hepatitis B virus infection (cHBV) affects millions worldwide. Inducing an effective T cell response is considered an essential component of a combinatorial treatment regimen to cure cHBV^28^. For this reason, we engaged in a study to design a novel HBV SLP-based therapeutic vaccine^11,29^. With this study we discovered components for a novel therapeutic vaccine. Recently, an SLP vaccine related to our work entered a phase I/II clinical safety and toxicity study (NCT05841095). In our studies we experienced that the design and testing of candidate SLPs and adjuvants relies heavily on in silico predictions and labor-intensive in vitro T cell assays^11,29,30^. Moreover, these in vitro assays are skewed towards HLA-II-mediated CD4 + T cell activation because of the scarcity of cross-presenting APCs in those cultures. A direct assay to measure SLP cross-presentation by DCs and the effect of adjuvants would thus greatly simplify and accelerate vaccine design and selection.

In this study, we utilize sensitive liquid chromatography tandem mass spectrometry (LC-MS/MS) to interrogate the immunopeptidome obtained from DCs matched on 2 common HLA-A alleles in the Asian and Caucasian population to find clinically relevant epitopes. DCs from 6 HLA-A*02:01/HLA-A*11:01 matched healthy donors were loaded with a prototype HBV vaccine consisting of 12 SLPs combined with either the TLR1/2 ligand AV (representing MyD88-dependent signaling) or TLR3 ligand PolyI:C (representing TRIF-dependent signaling). Although we did not find significant differences in the quantity nor quality (i.e., percentages of 8–12-mers and predicted binders) of the total DC immunopeptidome, including cross-presented SLP-derived peptides, we did identify a clear “TRIF” signature characterized by interferon-induced proteins. Furthermore, we show high efficiency of SLP cross-presentation and the sensitive and reproducible detection of SLP-derived peptides, including several established and potentially novel HBV CTL epitopes. Taken together, our work highlights the significant value of DC immunopeptidomics for vaccine design and antigen presentation research.

## Results

### DC antigen presentation does not quantitatively differ between adjuvants and is dominated by B alleles

**Fig. 1:**
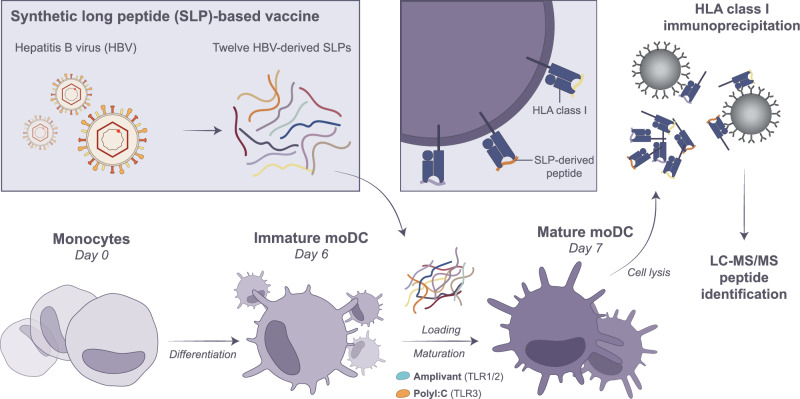
Schematic overview of moDC generation and SLP loading. Monocytes were isolated by plate-adhesion from healthy donor blood and differentiated with 500 IU/mL IL-4 and 800IU/mL GM-CSF for 6 days. On day 6, moDCs were pulsed with a cocktail of 12 SLPs (3 µM each) in combination with either TLR1/2 ligand Amplivant (3 µM) or TLR3 ligand Poly I:C (20 µg/mL) for 22 h. On day 7, moDCs were harvested and frozen as dry cell pellets until immunoprecipitation of HLA-I-peptide complexes. Eluted HLA-I peptides were subjected to DDA and DIA LC-MS/MS identification. This figure was created in Adobe Illustrator.

To inspect the pan-HLA-I presentation of SLPs by DCs, we loaded 12 previously designed hepatitis B virus-based SLPs^11^ (Sup. Table 1) on monocyte-derived DCs (moDCs) from 6 different healthy donors. Furthermore, to compare the impact of TLR signaling pathways on DC antigen (cross-)presentation, DCs were stimulated with either a TLR1/2 ligand (Amplivant; 3 µM) via the MyD88 signaling pathway, or a TLR3 ligand (PolyI:C; 20 µg/mL) via the TRIF signaling pathway (Fig. 1). Both TLR1/2 and TLR 3-based adjuvants are currently used in therapeutic vaccine regimens^30–34^. DCs were allowed to take up the cocktail of 12 SLPs in combination with these adjuvants for 22 h. Applied adjuvant concentrations were selected based on plateauing of maturation marker expression whilst maintaining cell viability (Sup. Fig. 1). Resulting DC phenotypes were similar between adjuvants with the exception of minor differences in CD14 and CD83 expression (Sup. Fig. 2).

HLA-I-peptide complexes were enriched by immunoprecipitation (IP) from frozen cell pellets with an overall average efficiency of 49.31% ± 17.08 (mean ± SD) across all samples, with no significant differences between adjuvants (Sup. Fig. 3A, B). In total, 34,315 unique peptides were confidently identified by LC-MS/MS data-dependent acquisition (DDA) over a total of 2 runs per sample, delivering 2,945 to 12,622 unique peptides per DC sample (7,044 ± 2,864; mean ± SD) (Sup. Fig. 4A). Total peptide numbers were not significantly different between the 2 adjuvants. On average, the dataset consisted of 93.2% ± 1.3% (mean ± SD) 8–11-mers, which were dominated by 9-mers, consistent with a typical peptide length distribution of HLA-peptides (Sup. Figs. 3C and 4B, C).

All donors were matched on HLA-A*02:01 and –A*11:01 alleles, but B- and C-alleles were variable across donors (Table 1). Of 8–11-mers, >93% was predicted to bind donor HLA for all samples (Sup. Fig. 4D). The number of HLA-binders was neither directly correlated to IP efficiency (as determined by Western blotting (WB)) nor to the *corrected cellular input* (CI); Sup. Fig. 3D). As before^35^, sample *HLA input* levels estimated from WB most associated to the number of HLA-binders, although not significantly (Sup. Fig. 3E–H, Sup. Table 2). As an additional quality control, scrambled peptides with the same length distribution and amino acid content were mapped back to donor HLA. Significantly less scrambled peptides mapped back, further indicating that our dataset is largely comprised of bona fide HLA peptides (Sup. Fig. 4E).

**Table 1:** Four-digit HLA typing of all donors included in this study

Healthy donor	A	A	B	B	C	C
D1	02:01	11:01	07:02	55:01	03:03	07:02
D2	02:01	11:01	35:01	40:01	03:04	04:01
D3	02:01	11:01	08:01	15:01	03:03	07:01
D4	02:01	11:01	35:01	40:01	03:04	04:01
D5	02:01	11:01	08:01	44:02	05:01	07:01
D6	02:01	11:01	07:02	51:01	07:02	14:02

Four-digit SNP HLA typing was performed to impute complete HLA profiles. All donors were matched on HLA-A*02:01 and HLA-A*11:01 expression.

Amino acid (AA) distribution analysis of all 8–11-mers across samples reflected the binding motifs of each sample^36^, and this did not differ between adjuvants (Sup. Fig. 5). As expected, the anchor residues at position 2 and the C-terminus were most pronounced in their AA preferences across all samples, whilst the middle “T cell receptor (TCR) recognition” region was most diverse. Despite the matching on both A-alleles, however, each donor displayed a distinctive AA distribution profile at the anchor residues that seemed to reflect mostly the B-alleles (Sup. Fig. 5b). This was especially apparent for donors that shared B-alleles (e.g. D1&D6 (B7:02), D2&D4 (B40:01), D3&D5 (B8:01) (Table 1)). D3 and D5 displayed the typical AA preference at positions 3–5 for the HLA-B8 super type, especially in shorter peptides. Concordantly, donors with overlapping B-alleles shared most peptides (Sup. Fig. 6).

Following up on this, we inspected the proportion of peptides predicted to bind to each of the classical HLA alleles (HLA-A, -B, and -C) for each donor (Sup. Fig. 4F). When we allowed peptides to be predicted to multiple alleles (i.e., ‘promiscuous allele mapping’), the majority of peptides was predicted to bind to the B- alleles, consistent with the observed AA distribution. Many peptides also predicted to bind to the C-alleles, and least were predicted to bind the A-alleles. To get more insight into the true origin of HLA-peptides, the analysis was repeated singling out the peptides that were predicted to bind only 1 out of 6 alleles (‘specific allele mapping’, Sup. Fig. 4G). Almost no peptides specifically binding C-alleles remained and peptides specific to the B-alleles outnumbered those specific for the A-alleles. Notably, subsequently assigning peptides to the allele with the highest rank score further indicated most peptides derived from (one of the) B-alleles (‘highest ranking mapping’, Sup. Fig. 4H) and to a lesser extent from the A-alleles. Taken together, there are no significant differences in moDC immunopeptidomes between adjuvants, and the moDC peptide repertoire is predominantly presented on B alleles.

### The DC immunopeptidome reflects ongoing cellular processes triggered by TLR stimulation

**Fig. 2:**
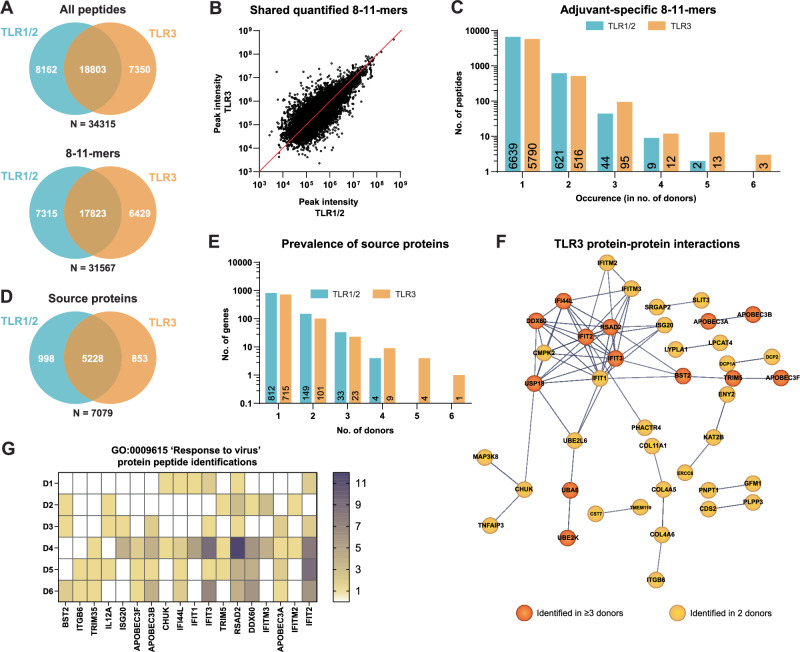
Ongoing cellular processes upon TLR stimulation are reflected by the immunopeptidome. **A** Overlap of all peptides (upper) and 8–11-mers (lower) between TLR1/2 and TLR3 peptide datasets. **B** Correlation plot of the peak intensities that could be inferred for shared peptides between TLR1/2 and TLR3 datasets. **C** The occurrence rate of all adjuvant-specific 8–11-mers within the pool of 6 donors. **D** Overlap in source genes between TLR1/2 and TLR3 8–11-mer datasets. **E** The occurrence rate of adjuvant-specific source genes within the pool of 6 donors. **F** Protein interaction network as determined by STRING analysis for all TLR3-specific source proteins identified in ≥3 out of 6 donors (orange). The network was further supplemented by TLR3-specific source proteins identified in 2 out of 6 donors (yellow). Confidence level: 0.7. **G** The identification frequency across 6 donors of 18 TLR3-specific source proteins contained within the top-hit Gene Ontology biological process ‘Response to virus’. Frequency values as stated in the color scale are based on the amount of unique peptide sequences derived from each source protein. White = no identifications.

Having established the quality and HLA-origin of the peptidome, we then questioned whether the content of the immunopeptidome was different between TLR1/2 and TLR3 matured moDCs. Fifty-five percent of identified peptides were shared between adjuvants and also identified with similar peak intensities (Fig. 2A, B). Conversely, 45% of all identified peptide sequences were uniquely identified in DCs matured using one or the other adjuvant. Of those unique peptides, however, >98% was detected in the DC immunopeptidome of 1 to 2 out of 6 donors only and hence likely reflected experimental and/or biological variation rather than a true effect of differential TLR stimulation (Fig. 2C). Compared to TLR1/2, TLR3 stimulation yielded more adjuvant-specific peptides (55 versus 123 peptides in total).

Because more than one HLA-peptide can be derived from each protein binding to different HLA-alleles, overlap between samples might be higher when considering the source proteins of the HLA-types. Hence also TLR-specific effects could be more apparent at the protein level. Zooming out to the source proteins, 14% (998) and 12% (853) of proteins giving rise to HLA-peptides were unique to TLR1/2 and TLR3 stimulation, respectively (Fig. 2D). A few, mostly TLR3-unique, source proteins were presented in multiple donors (Fig. 2E), rendering these more robustly associated with adjuvant-specific signaling. Utilizing STRING and Gene Ontology (GO) analysis, we aimed to uncover any meaningful interactions between adjuvant-specific source proteins, and represented biological processes. For the TLR3-specific (but not TLR1/2-specific) source proteins, inter-protein connectivity was significantly higher than expected (4.7-fold higher, *p* < 1.0e-16). The most significantly upregulated GO biological process was ‘Response to virus’, consistent with the role of TLR3 in the sensing of RNA fragments (Fig. 2F, Sup. Table 3). Source proteins uniquely delivering peptides to the DC immunopeptidome of 3 or more donors made up the core of the interaction network and reflected the TLR3/TRIF-induced maturation of DCs. This network could further be extended by including those proteins delivering adjuvant-specific HLA-peptides in 2 out of 6 donors. Notably, 18 TLR3-specific source proteins affiliated with the GO term “response to virus” were accounted for in the immunopeptidome of multiple donors (Fig. 2G). In particular, peptides from interferon-induced proteins IFIT2, IFIT3 and RSAD2 were identified in samples from all but 1 donor or even all, respectively. Cumulatively, this suggests that the obtained DC immunopeptidome reflects ongoing biological processes in these cells and that applied immunopeptidomics based on DDA LC-MS/MS workflow is sufficiently sensitive to accurately capture meaningful differences in the immunopeptidome.

### Highly reproducible identification of cross-presented SLP peptides by DC DDA LC-MS/MS irrespective of adjuvant

Next, we evaluated the cross-presentation of SLPs by moDCs and to assess whether DC immunopeptidomics can be used to identify which SLP-derived peptides are cross-presented on HLA-I in a T cell-free assay. Such information would be highly valuable to select both SLPs and adjuvants for therapeutic application. Using DDA, 33 unique SLP-derived HLA-peptides were identified in total from 10 out of 12 SLPs. As many as 26 peptides were reproducibly identified in 2 or more samples (Table 2). Moreover, we discovered 14 novel HBV-derived HLA-peptides. No peptides with amino acid sequences matching any of SLPs were identified in matured DCs in the absence of SLPs (data not shown). All SLP-related peptide spectrum matches (PSM) were manually inspected and 27 out of 33 spectra were determined to be of medium (12) to good (15) quality (Table 2, Sup. Fig. 7; See methods for quality criteria). The quality of the remaining 6 spectra was poor. Yet, 4 of these were predicted to bind HLA types expressed in all the DC samples of origin, indicating these still may be bona fide HLA-presented peptides (Table 2)^37,38^.

**Table 2:**
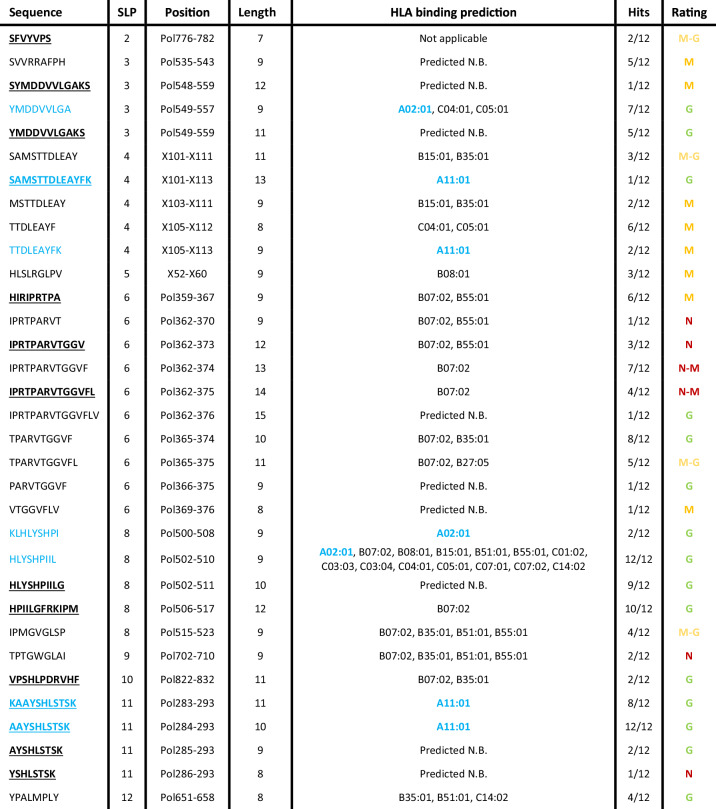
Overview of all identified SLP peptides

Thirty-three unique SLP peptides were identified by DDA LC-MS/MS. Given from left to right are: peptide sequence, SLP of origin, canonical protein position, peptide length, HLA binding prediction by NetMHCPan4.1 for all donor HLA alleles within the dataset, the number of hits across all samples (12 total), and the quality rating of the PSM. *N* not good, *M* medium, *G* good. ‘Not applicable’ = HLA binding prediction could not be performed due to a length shorter than 8 amino acids. ‘Predicted N.B. (non-binder)’ is stated when a peptide was ranked >2.0%. All peptides predicted to bind HLA-A*02:01 or HLA-A*11:01 are highlighted in blue. All sequences bold and underlined are never before reported.

In vivo, primary DCs may also play a role in SLP cross-presentation. Our effort to also obtain the immunopeptidome of primary DCs from buffy coats was not yet producing up-to-standard immunopeptidomes (Sup. Fig. 8). Peptide numbers and peptidome quality were evidently less than for moDC samples. Yet, 9-mer peptides were most prevalent in all samples and 2 SLP-derived peptides were identified (SFVYVPS in D7&D9, TTDLEAYF in D7), indicating the near-feasibility of HLA-I immunopeptidomics on primary DCs obtained from large blood samples.

In DDA mass spectrometry, peptide precursors are selected for fragmentation based on their relative spectral intensities and, therefore, there is a bias towards the more abundant peptides in a complex sample. In contrast, Data Independent Acquisition (DIA) mass spectrometry might offer a more comprehensive view on peptide presence, since all peptide precursors are selected and fragmented simultaneously, including the very low abundant ones^39^. Hence, in an effort to further extend the existing DDA-based dataset, we explored the use of DIA-based LC-MS/MS and the remaining part of the moDC samples was subjected to a DIA-based LC-MS/MS analysis (Sup. Fig. 9). Although this single DIA run resulted in a lower number of identified peptides than the 2 previous DDA LC-MS/MS runs combined (i.e. 22,070 peptides), the expected length distribution that is typical for HLA-I peptides was also observed. Moreover, DIA-based LC-MS/MS uncovered one new SLP-derived peptide sequence (VVLGAKS) and also expanded the sample coverage for several peptides. The moDC DDA-based method, however, produced the most informative and complete dataset, and we continued by interrogating it more deeply.

**Fig. 3:**
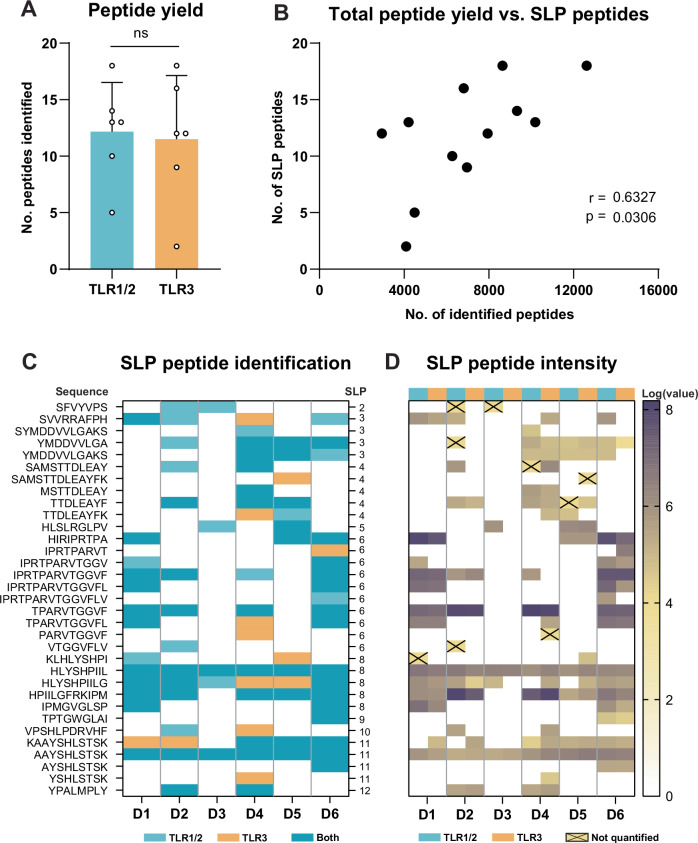
Thirty-three unique peptides from 10 SLPs are reproducibly identified in 6 donors with DDA LC-MS/MS. **A** The number of distinct SLP-derived peptides per sample. Two-tailed paired T-test. ns = non-significant. **B** Correlation plot of the number of identified SLP peptides and total peptide yield. Two-tailed non-parametric Spearman’s rank correlation. **C** Overview of SLP peptide identifications. Given are the peptide sequence, identification per donor and SLP of origin. **D** Log-transformed peptide signal intensities per SLP peptide identification. Signal intensity values of identifications annotated with a cross could not be quantified by Peaks.

The moDC DDA-based analysis yielded up to 18 distinct SLP-derived peptides per sample. Despite our ability to detect differences in used signaling routes from the overall immunopeptidome, the number and identity of SLP-derived peptides did not significantly differ between DCs matured with TLR1/2- and TLR3-based adjuvants, indicating adjuvants support SLP cross-presentation similarly (Fig. 3A). As expected, there was a significant correlation between the size of the total immunopeptidome and the number of SLP-derived peptides identified per sample, indicating that the identification sensitivity of SLP-derived peptides depends on sample coverage (Fig. 3B). Some SLPs yielded one peptide, whereas others yielded 5 or more with overlapping sequences. Furthermore, the majority of SLP-derived peptides was identified in more than 1 donor (Fig. 3C). One of two peptides identified in all samples was the SLP8-derived peptide HLYSHPIIL, a previously described HLA-A2 epitope derived from the HBV polymerase protein^29,40^. Interestingly, the 1 amino acid longer peptide, HLYSHPIILG, was nearly as often identified, yet was not predicted to bind any of the DC expressed HLA-types (Table 2). Additionally, an entirely novel polymerase-derived peptide AAYSHLSTSK (SLP11, assigned to HLA-A11) was identified across all donors. Next, as a rough estimate of peptide abundance, all signal intensities of the spectra matched to SLP-derived peptides were interrogated (Fig. 3D). From 10 out of the 166 identifications of SLP-derived peptides, no signal intensities could be inferred by the software (crossed boxes). As expected, the most reproducibly found SLP peptides predominantly were relatively highly intense in the spectra. Taken together, whilst moDC DDA-based LC-MS/MS did not find any effects of adjuvants on the cross-presentation of exogenously offered SLPs, it did successfully uncover a significant number of HLA-peptides from these SLPs; including several repetitively identified known and novel potential T cell epitopes.

### HLA presentation of SLPs by DCs cannot be fully predicted in silico

**Fig. 4:**
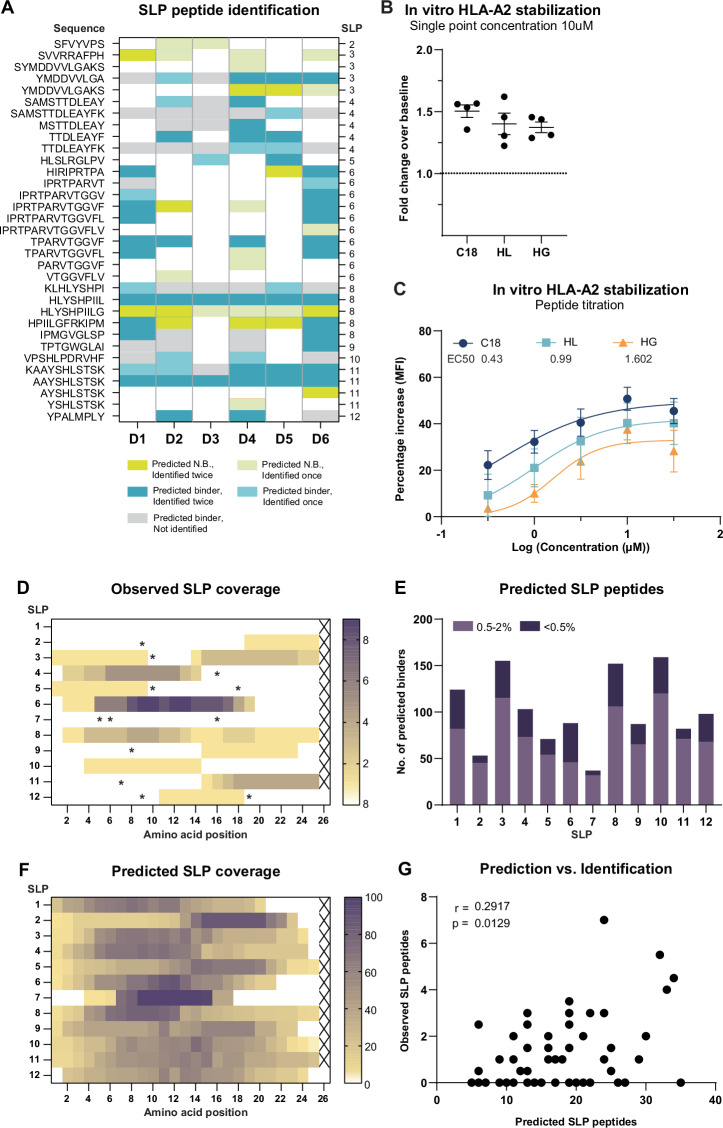
HLA-restricted SLP cross-presentation by moDCs is not yet accurately captured by in silico prediction algorithms. **A** An overview of NetMHCPan4.1-predicted HLA binding for identified SLP-derived peptides (≤2.0% rank). Given in lime green are positive identifications that were predicted non-binders; in blue positive identifications of predicted binders; in gray predicted binders not identified within this data-set. **B** HLA-A2 stabilization as measured by the T2 assay by HLYSHPIIL and HLYSHPIILG at a concentration of 10 µM. The HBcAg18–27 epitope was taken along as a positive control, DMSO was taken along as a negative background control (baseline). **C** All three peptides were titrated by incubation of T2 cells with 0.3, 1, 3.1, 10 and 31 µM peptide. Lines correspond to the fitted binding curves calculated by non-linear regression with the method of least squares. **D** Cumulative coverage of SLPs by identified SLP-derived peptides in all samples. Color intensity is determined by the identification frequency of each AA position. Asterisks indicate presence of cysteines. **E** Number of NetMHCPan4.1-predicted binders contained within each SLP for all 6 donors’ HLA-alleles. Weak binder (purple): rank 0.5–2%, strong binder (dark purple): rank ≤0.5%. **F** Cumulative predicted coverage of SLPs based on NetMHCPan4.1 peptide prediction across all 6 donors. Color intensity reflects the percentage of AA coverage relative to total coverage by all predicted peptides for each SLP. **G** Correlation between the number of observed SLP peptides and the number of predicted SLP peptides per donor, per SLP. Two-tailed non-parametric Spearman’s rank correlation.

We did not identify peptides from all SLPs, and some SLPs yielded many more peptides than others. To find an explanation for the variable presentation of the different SLPs, we next investigated the relationship between MS identification and in silico prediction. Most often, SLP-derived peptides were predicted to bind either the B or the C alleles, and hence were not expected to be identified in all donors as these were only matched on the A-alleles (Table 2, Sup. Table 4). Despite this matching, the MS identification of predicted HLA-A*02:01 and HLA-A*11:01 binders was also variable; some, including the dominant A2 epitope YMDDVVLGA, A2 epitope HLYSHPIIL and potential novel A11 epitope AAYSHLSTSK were identified across nearly all donors. Others, such as novel A11 binder SAMSTTDLEAYFK, A11 epitope TTDLEAYFK, and A02 epitope KLHLYSHPI, were only identified in 1 or 2 donors (Fig. 4A). Of note, donors 4 & 6, and donors 1 & 8 shared a high number of SLP peptide identifications, which was explained by the fact that these donors shared 6 and 4 HLA alleles, respectively (Table 1, Sup. Fig. 9). Intriguingly, one of the most reproducibly MS-identified SLP peptides (HLYSHPIILG) was not predicted to be a binder (Fig. 4A, Table 2). Because of its close relation to the established HLA-A*02 epitope HLYSHPIIL, we postulated HLA-A*02 to be the most likely source of this peptide. To test this hypothesis, we subjected both peptides (i.e. HLYSHPIIL and HLYSHPIILG) to an T2 cell-based HLA binding assay^41^. Although with somewhat lower capacity than HLYSHPIIL, HLYSHPIILG bound to HLA-A2. Notably, at 10 µM, HLYSHPIILG (and HLYSHPIIL) displayed equal ability to stabilize cell surface HLA-A2 as compared to the established HBcAg18–27 epitope that is considered to be a very strong binder (Fig. 4B, C).

Extending on the comparison between observed and predicted SLP-derived peptides, we mapped the observed coverage for each SLP. Out of the 12 SLPs loaded onto the DCs, most were derived from SLP6, yielding a total of 11 distinct peptides that all derived from the same “hotspot” within the SLP (Fig. 4B). The latter in contrast to SLP 8, that yielded 6 unique peptides that covered the entire stretch of 25 amino acids but 1. From SLP 2, 5, 9, 10 and 12 only 1 peptide sequence could be identified. No peptides were identified from SLP 1 and 7. Interestingly, across SLPs, no peptides were identified from cysteine-containing regions, known to interfere with MS identification (Fig. 4D, asterisks)^42^.

Finally, to then compare the identified coverage to the in silico predicted coverage of the SLPs, each SLP was interrogated for the number of predicted HLA-binding peptides for all donors’ HLA (Fig. 4E), which were also plotted in a heat map (Fig. 4F). SLP 2 and 7 were predicted to yield the least HLA-binders. Concordantly we only identified 1 and 0 peptides from these SLPs, respectively. SLP 6 was among several SLPs predicted to contain many high affinity peptides, which may partially explain the large number of HLA-binders identified across all donors. The correlation between predicted HLA-binder content and MS peptide recovery was significant but varied per SLP (Fig. 4E–G). Especially for SLPs 2, 4, and 6 there was a good consensus between predicted and observed peptide generation, whereas for other SLPs (e.g. SLPs 1 and 10) this was less apparent. Taken together, predicted HLA-binder content not fully explained the variable recovery of peptides from the 12 different SLPs, underscoring the added value of MS analysis of SLP-loaded DCs.

## Discussion

For the design of next-generation SLP-based therapeutic vaccines it is crucial to know which SLP-derived CTL epitopes are effectively cross-presented by DCs^18,43^. We here describe the most exhaustive DC HLA-I ligandome study on SLP-loaded HLA-A-matched DCs to-date. In this study, we thoroughly interrogate both the cross-presentation of 12 HBV-based SLPs and the effect of 2 vaccine adjuvants on antigen (cross-)presentation. In DC HLA-eluates we have identified 33 HBV SLP-derived HLA-peptides, including several (well) known epitopes and 14 novel potential HBV T cell epitopes, most of which we detected with high reproducibility and confidence. Although our analyses have demonstrated that the nature of the TLR ligand used for DC maturation could be deduced from the full immunopeptidome, we did not find a significant difference between adjuvants in their support of SLP cross-presentation. With this work, we add valuable insights to the field of DC antigen presentation and demonstrate that DC immunopeptidomics represents a valuable tool directly applicable to study the regulation of DC antigen presentation as well as therapeutic vaccine design.

Choice of adjuvant determines the skewing of a vaccine-induced immune response. We have previously compared Amplivant® (TLR1/2) and PolyI:C (TLR3) for their ability to support DC cross-presentation of an SLP-contained HBV epitope (HBc_18-27_) to cognate CD8 + T cells in vitro^30^. Although both adjuvants facilitated cross-presentation of HBc_18-27_ in vitro, it was difficult to discern whether these adjuvants differentially affect SLP processing and cross-presentation. Firstly, the applied T cell-based assay considered only one SLP-contained CD8 + T cell epitope, and secondly, in such assay it is hard to determine the differences in adjuvant-induced effects on HLA presentation as results are confounded by effects on the expression of co-stimulatory molecules and cytokines. We have now obtained a more comprehensive view on SLP cross-presentation by directly interrogating the immunopeptidome of SLP-loaded DCs.

Obtained data indicate that the use of a TLR1/2- versus a TLR3-based adjuvant does not qualitatively affect SLP cross-presentation. The minor but consistent difference in the DC immunopeptidome between adjuvants reflected the known action of TLR3 via TRIF on anti-viral pathways^23^, and hence was likely the result of direct co-translational HLA-loading via the proteasome/ER of peptides derived from TRIF-signaling-induced proteins. Concordantly, with the notion that both TLR1/2 and TLR3 signal via TRAF6 to induce e.g. NF-KB, no TLR1/2-unique immunopeptidome was observed. Our in vitro study does not exclude that in vivo SLP antigen (cross)presentation might still be shaped by the variable action of adjuvants on different conventional DC (cDC) subsets. While moDCs express both TLR1/2 and TLR3, in vivo TLR3 is only expressed on cDC1, which are deemed better at cross-presentation. In contrast to TLR3, TLR1/2 is more widely expressed on cDC subsets in vivo^44,45^. Ultimately, to target the antigen and adjuvant to the same DC, conjugation of adjuvants to SLPs or co-encapsulation in the same particle may be needed^30,31,46^. TLR signaling from phagosomes has been demonstrated to regulate antigen presentation at the organelle-autonomous level^47^. Therefore, it is possible that upon association of SLP and TLR ligands, intracellular trafficking and/or endosome maturation, and thereby cross-presentation, might still be different for TLR1/2 and TLR3-based adjuvants^48^. Despite these limitations, the identification of a “TRIF” signature in our study does demonstrate DC immunopeptidomics is sufficiently sensitive and reproducible to detect differences between variably treated samples. Hence, DC immunopeptidomics may be used as a tool to study how drugs and adjuvants affect DC antigen (cross-)presentation.

Our dataset of >36,000 unique HLA-I peptides from 6 HLA-A-matched donors allowed us to investigate how the different HLA genes and alleles shape the DC immunopeptidome. The AA distribution reflected matching on HLA-A2:01 and HLA-A11:01 alleles by the consistent presence of xLxxxx(x)_1-4_L/V or xVxxxx(x)_1-4_K motifs in each sample respectively. Like others^49^, we found a correlation between the number of shared HLA alleles and peptides. Importantly, two donors within our dataset that uniquely shared all classical HLA-I alleles also displayed a near identical peptide AA distribution, reflecting both the high robustness of our method and the strong imprinting of HLA types on the immunopeptidome. Between these donors almost half of the DC immunopeptidome was shared, indicating HLA-I presentation is highly conserved between individuals expressing the same HLA alleles.

For the other samples, the AA distribution was still surprisingly variable between most donors despite the match on both HLA-A alleles, suggesting a significant contribution of also the B and/or C-alleles to the DC immunopeptidome. Indeed, most HLA peptides mapped back to the A- and B-alleles rather than to C-alleles, pointing towards a dominant impact of these alleles and especially the B-alleles on the DC peptide repertoire. This is consistent with the notion that HLA-A and -B expression is generally several folds higher than HLA-C^50,51^, and aligns well with moDC immunopeptidome data by others^52^. Additionally, of the cross-presented peptides, 18 out of 25 predicted binders (72%) were assigned to B-alleles, although this might have been a result of more variation on the B alleles due to preselection of the A alleles. Among these, many were predicted to bind B-alleles particularly highly expressed by monocytes^53^ (e.g. B*07:02 and B*35:01, predicted to bind 12 and 7 MS identified peptides respectively). Some B alleles, such as B*35:01, might be particularly receptive for cross-presentation because of a higher stability in the endolysosome, allowing for more efficient loading of cross presented peptides in this compartment^50^. Considering the dominance of the B-alleles in DC antigen-(cross)-presentation, HLA-B epitopes may be of particular interest for vaccine design.

Our dataset also provided the opportunity to assess the performance of in silico prediction for cross-presentation. The mean percentage of predicted binders for all MS-identified 8–11-mers was 93%, as opposed to only 74% for MS-identified SLP-derived peptides. Possibly the NetMHCpan4.1 algorithm, trained on HLA-I peptides derived from mostly parenchymal (cancer) cells rather than DCs^54^, can less accurately predict binding of cross-presented peptides because DCs have a more complex antigen processing machinery. Whereas in parenchymal cells the generation of endogenous HLA-I peptides is (immuno)proteasome-driven, the generation of exogenous peptides for cross-presentation by DCs is a combined action of the (immuno)proteasome as well as endosomal proteases with very different cleavage specificities^55–57^. This is exemplified by the identification of SLP-derived peptides HLYSHPIIL (Pol_502-510_) and HLYSHPIILG (Pol_502-511_). HLYSHPIIL but not HLYSHPIILG is a predicted HLA-A2:01-binder and demonstrated HLA-A2:01 epitope^11,58,59^. Both peptides are reproducibly found to be presented across all donors with high confidence (i.e. with high quality spectra) and using an in vitro HLA-binding assay we confirmed the binding of both peptides to HLA-A*02:01. The addition of the glycine at the C-terminus, however, renders the peptide a predicted non-binder to HLA-A*02:01, which is in line with the notion that glycines are rarely found at the last position of HLA-I-eluted peptides^36,60^. Likely HLYSHPIILG is the product of endosomal cathepsins rather than of the (immune) proteasome and for this reason was not predicted as a binder for HLA-A2:01^61,62^. Structure-based modeling suggests that the binding of HLYSHPIILG to HLA-A*02:01 is, similar to its shorter counterpart, likely based on the leucine at P9^63^. This suggests that the T cell-exposed surface of the HLYSHPIIL-HLA-A2:01 and HLYSHPIILG-HLA-A2:01 are highly similar, although it remains to be determined whether they are indeed similarly recognized by the same TCRs.

Nonetheless, our data suggest that presentation of HLYSHPIIL(G) by DCs is highly conserved across individuals, rendering it a highly potent epitope as efficient cross-presentation by DCs is associated with T cell dominance^18^. Importantly, HLYSHPIIL drives CTL-mediated killing of HepG2.2.15, a hepatoma cell line overexpressing the HBV genome^40^, indicating this peptide can also be loaded on HLA via the direct co-translational route of antigen presentation, as would occur in HBV-infected cells. Although HLYSHPIIL has already been proposed as a promising therapeutic target for HBV vaccines, it is not nearly as well-established as e.g. the HBcAg_18-27_ HBV epitope^29,40^. Our data, together with existing literature, suggest it may be worthwhile to revisit Pol_502-510(511)_ as a high potential immunodominant HLA-A*02:01 epitope to target with therapeutic vaccination. Intriguingly, Pol_502-510_ is predicted to bind many other B and C alleles. Although this prediction needs validation this indicated Pol_502-510_ may have potential beyond HLA-A2 as a “universal” HBV CTL epitope.

The therapeutic potential of SLPs has already been clinically established for multiple indications and has been shown to induce broad CD4+ and CD8 + T cell responses^3,4,11,14–16,30,64^. Information on the cross-presentation efficiency of candidate SLPs across donors, or even per donor, could aid the selection of SLPs for clinical testing, vaccine personalization, and/or prediction of responses^11^. As such, the data presented here is of direct interest for the further development and refinement of an HBV SLP-based therapeutic vaccine.

In total, of the 12 tested HBV-antigen based vaccine candidate SLPs, we identified 35 different SLP-derived peptides from different 10 SLPs across 6 donors. No peptides derived from SLP1 (Pol_140-164_) and SLP7 (X_2-26_) were detected. It is generally assumed that mass spectrometry-based HLA peptide identification assays produce representative patterns of peptides that are HLA-presented. However, it cannot be excluded that (a subset of) HLA-presented peptides are not responsive in electrospray ionization-based LC-MS/MS and may, thus, not be measured. Additionally, the spectral intensities of detected peptides may not necessarily reflect the relative abundances of those peptides in the biological sample. Looking at other explanations for their absence, SLP7 contains the lowest number of predicted epitopes and contains 3 cysteines throughout its sequence, potentially hampering LC-MS/MS detection^65^. In vitro this SLP was able to activate T cells from HBV exposed individuals but, in contrast to the other tested SLPs and consistent with its here observed inefficient cross-presentation, it induced CD4 + T cells responses only^11^. The reason for the absence of SLP1 (HBV Pol_140-164_)-derived peptides in the DC immunopeptidome remains unexplained but was also in line with a low number of HBV resolver T cells responding to this SLP in vitro^11^. Especially SLP8 (containing the HLYSHPIIL(G) epitope) was cross-presented well across all donors, and also induced broad CD8 + T cell responses in both HBV resolvers and chronic patients^11^, rendering it a prime vaccine candidate. Also several other SLPs (e.g. 11, 6, 4 and 3) were cross-presented by DCs from most donors and are therefore also of interest to include in a multi-SLP vaccine cocktail. Especially SLP4 and 11 are of interest as these also contain established and potential novel epitopes for HLA-A*11:01, which is prevalent in the Asian population that is highly affected by chronic HBV^66^.

We here present a pipeline that is robust and easy to implement. Furthermore, we demonstrate that it provides the opportunity to scrutinize the presentation efficacy of multiple potential vaccine candidates at once. Notably, we have obtained sizable immunopeptidomes of on average 6E3 peptides (and identified ≈10 SLP-derived peptides) from on average 15E6 moDCs as starting material. This numbers of cells is 1–2 orders magnitude less than typically required from other cell types to achieve similar results^67^. This number of moDCs can be easily obtained from small volume blood samples^68^. The lower sample requirement likely stems from the high HLA expression on (mo)DC^68^. Despite that moDC are an in vitro generated experimental model, the obtained data and pipeline are clinically relevant as in vitro generated moDC resemble inflammatory DCs that arise in pathogenic situations in vivo^69^. Furthermore, in vitro generated moDCs loaded with peptides mRNA or tumor lysates are currently directly applied as therapeutic vaccines for a multitude of cancers^8^. As such, it would be highly interesting to apply our pipeline to DCs loaded with tumor lysates to uncover the most important tumor associated antigens presented by such vaccine preparations. Comparing these results to antigen expression or presentation in recipients’ tumors could then aid selection of patients that would benefit from tumor lysate loaded moDC vaccination. Similarly testing an SLP or mRNA vaccine on patients’ DC prior to vaccination might predict which patient may benefit from the vaccine.

To broaden translational value, vaccine antigen cross presentation by moDCs would benefit from direct comparison to primary cDC. Although not yet up-to-standard, we demonstrated the near feasibility of immunopeptidomics on primary DCs and identified 2 SLP-derived peptides in the HLA eluate of primary DCs which were also found on moDCs. As technology advances and sensitivity increases, patient-derived primary DC may in the future be pulsed with vaccine antigens to predict patient responses and/or to stratify patients into different vaccine groups.

Taken together, our study brings novel insights into DC antigen presentation that are of direct therapeutic relevance for the development of antigen-based therapies. DCs are at the center of a potent effector CTL responses and therefore, when developing vaccines, it is pivotal to uncover what DC present on HLA-I. Ultimately, we envision DC immunopeptidomics can serve a valuable tool to speed up, direct, improve and patient tailor vaccine design.

## Methods

### Generation and synthetic long peptide loading of dendritic cells

Twelve HBV SLPs (Sup. Table 1) were gifted by ISA pharmaceuticals and designed and synthesized as described before^11^. Peripheral blood mononuclear cells (PBMCs) were isolated by Ficoll-Paque (Merck) density gradient centrifugation from buffy coats of HLA-A*02:01 + HLA-A*11:01+ healthy donors collected from the local blood bank (Sanquin, The Netherlands). Buffy coats constitute leftover material and no ethical approval is required for use. Written informed consent for use was obtained from all donors. Thrombocytes were removed by stringent washing (4-5x) with room temperature (RT) PBS (Fisher Scientific), 2 mM EDTA (Westburg), 0.1% BSA (Sigma-Aldrich), followed by a final wash with Iscove Modified Dulbecco Medium (IMDM; Fisher Scientific) + 2 mM UltraGlutamin (Lonza) to remove remnant EDTA. For the generation of monocyte-derived dendritic cells (moDCs), PBMCs were seeded out in T75 flasks (Corning) at a concentration of 12.5 million cells/mL in cold IMDM containing penicillin/streptomycin (P/S; Life Technologies Europe BV) and 2 mM ultraglutamine (adherence medium) to facilitate monocyte adherence as before^21^. After 1 h incubation at 37 °C, 5% CO_2_, non-adhered peripheral blood lymphocytes (PBLs) were removed from the flasks by vigorous tapping and washing with PBS (RT). PBLs were frozen in IMDM with 10% DMSO (Sigma Aldrich) and 45% foetal calf serum (FCS; Gibco) for later use. Differentiation medium (IMDM supplemented with 8% FCS, 1% P/S, 2 mM UltraGlutamin, 500 U/mL IL-4 and 800 U/mL GM-CSF (Peprotech)) was added to the adhered monocytes cells to induce differentiation into moDCs for 3 days at 37 °C, 5% CO_2_. Differentiation medium was replenished on day 3 for a further 3-day differentiation. On day 6, the medium was replaced with medium containing the 12 SLPs (3 µM each; ISA Pharmaceuticals) and either TLR3 ligand PolyI:C (20 µg/mL; InvivoGen) or TLR1/2 ligand Amplivant® (3 µM; ISA Pharmaceuticals) for 22 h. Primary DCs were enriched from PBMCs by magnetically activated cell sorting using the Pan-DC Enrichment Kit (Miltenyi). Manufacturer’s instructions were followed. Enriched primary DCs were seeded into 6-well flat bottom plates at a concentration of 2 × 10^5^ per well. Primary DCs were stimulated and loaded with either TLR3 ligand PolyI:C (20 µg/mL) or TLR1/2 ligand Amplivant® (3 µM) and the 12 SLPs (3 µM each) for 16 h. After incubation, loaded DCs were harvested with cold PBS, counted, and frozen as dry cell pellet until further use.

### Generation of pan-HLA-I protein A sepharose beads

The anti-pan-HLA-I antibody W6/32 was produced in-house from hybridoma cells (HB-95, ATCC). Using a P1 peristaltic pump (GE Healthcare)-driven column set-up, the W6/32 antibody-containing medium was pumped over nProtein A sepharose Fast Flow beads (Cytiva) at a concentration of 3.2 mg W6/32 antibody per mL packed beads to create pan-HLA-I affinity beads. Antibody crosslinking to beads was performed with 16 mM dimethyl pimelimidate dihydrochloride (Sigma Aldrich) in 0.2 sodium borate buffer pH 9.0 for 30 min at RT on a rollerbank, before being quenched with 0.2 M ethanolamine pH 8.0 for 1 h at RT on a rollerbank. Conjugated pan-HLA-I affinity beads were washed twice with 0.1 M glycine pH 2.5, followed by three washes with RT PBS. Affinity beads were stored as a 50% slurry in PBS + 0.1% sodium azide at 4 °C until use.

### Enrichment of HLA-I-peptide complexes

Based on a previously published protocol, frozen dry pellets were resuspended on ice in cold cell suspension buffer (CSB; 50 mM Tris-HCl pH 8, 150 mM NaCl, 5 mM EDTA, 1 tablet protease inhibitor (Roche)/50 mL) to a concentration of 37 × 10^6^ cells/mL in LoBind tubes (Eppendorf)^70^. Cells were further diluted 1:1 with ice-cold lysis buffer (1% N-dodecyl-N,N-dimethyl-3-ammonio-1-propanesulfonate (Zwittergent, Merck) in CSB) to 18.5 × 10^6^ cells/mL and incubated on ice for 1 h whilst periodically vortexed every 15 min. Cell lysates were cleared from debris by 10 min centrifugation at 13.3 × g, 4 °C. Resulting supernatant (termed post-nuclear supernatant, PNS) was used for HLA-I immunoprecipitation (IP). Following a previously published protocol, mini-columns were produced by the insertion of half of a P20-filter into a P1000 tip with the cut-side down^70^. Filters were rinsed with 1 mL PBS prior to the application of either unconjugated protein A Sepharose CL-4B beads (Cytiva) for the pre-clear column or conjugated anti-pan-HLA-I W6/32 beads into the affinity column. Beads were washed once with washing buffer (0.5% Zwittergent in CSB) before PNS was sequentially run once across the pre-clear column and five times across the affinity column at a flow rate of 1 drop per second. The affinity beads were sequentially washed as follows: thrice with washing buffer, twice with Tris-120mM NaCl, once with Tris-1M NaCl, twice with Tris-120mM NaCl, once with PBS-20mM Tris-HCl pH8, and once with PBS.

### Extraction of HLA peptides and liquid chromatography tandem mass spectrometry analysis

HLA peptides were eluted from the beads by adding 400 µl 0.15% TFA at room temperature. This procedure was repeated 3 times to ensure collection of all peptides. To separate the HLA peptides from proteins, the extracted HLA peptides were filtered by centrifugation on 10 kD MWCO columns (Microcon-10, MRCPRT010, Millipore). The filtered peptide fraction was desalted on an in-house made 1cc Sep-Pak column containing 10 mg C18 and 10 mg HLB resin. Peptides were eluted with 28% Acetonitrile with 0.1% TFA and dried in a vacuum centrifuge. Nanoflow liquid chromatography tandem mass spectrometry (LC-MS/MS) was performed on an EASY-nLC 1200 coupled to an Orbitrap Eclipse Tribid mass spectrometer (Thermo) operating in positive mode. Peptide mixtures were trapped on a 2 cm × 100 μm Pepmap C18 column (Thermo Fisher) and then separated on an in-house packed 50 cm × 75 μm capillary column with 2.4μm Reprosil-Pur C18 beads (Dr. Maisch) at a flowrate of 275 nL/min, using a linear gradient of 0–32% acetonitrile (in 0.1% formic acid) during 120 min. MS spectra were acquired from 375 to 1200 m/z in the Orbitrap with 120 K resolution. Peptides were fragmented by HCD with a collision energy of 30% and MS/MS spectra were recorded in the Orbitrap with 30 K resolution.

### LC-MS/MS data analysis

MS/MS spectra obtained by data-dependent acquisition (DDA) and data-independent acquisition (DIA) were searched in PEAKS Studio (v11.0, Bioinformatics Solutions Inc.) against a database including the human reference proteome (UniProt, UP000005640, 02 Mar 2022), 4 HBV reference proteomes (UP000008591, UP000121470, UP000007930, UP000172538) and the 12 SLP sequences (Sup. Table 1). The false discovery rate was set to 0.05^37^. Data were processed by an in-house developed R-script using the Tidyverse^71^, Glue^72^ and Conflicted^73^ packages. In brief, sample annotations were first added to the PEAKS output data before dealing with equivalent PSMs (i.e. calculation of signal intensity values in the case of PSM replicates). All possible isoleucine/leucine (I/L) duplicates were identified and flagged (Sup. Data 1), but not excluded from the data analysis as to not to introduce any bias. The length distribution and amino acid distribution were generated without excluding possible isoleucine/leucine duplicates. Each PSM was annotated to a single source protein before exporting data into Excel. HLA mapping was performed on all unique sequence 8–11-mer peptides for each sample by using NetMHCpan4.1^54^. Peptides with a binding rank ≤2.0% were annotated as binders. Prediction of possible HLA binders from each SLP was also performed in NetMHCpan4.1 using a binding rank threshold of 0.5%- ≤ 2.0% for weak binders and ≤0.5% for strong binders. The predicted SLP coverage was determined by running each SLP through the NetMHCpan4.1 prediction algorithm for each donor separately. Predicted peptides for all donors were then combined for each SLP. Coverage of each amino acid within the SLP sequence was calculated based on the number of times that that amino acid position was contained within the predicted peptides across all donors. This cumulative coverage number was then calculated as a percentage of the total amount of predicted peptides and visualized in a heat map. The mass spectrometry proteomics data have been deposited to the ProteomeXchange Consortium via the PRIDE^74^ partner repository with the dataset identifier PXD051490.

### Quality of SLP-related peptide spectrum matches

All SLP-related peptide spectrum matches were manually inspected for quality control. Spectra were categorized as ‘*good*’ if both the number of fragment ion peaks, their signal-to-noise ratios and the completeness of the fragment ion series were substantial. Spectra were categorized as ‘*medium*’ if only a part of the b or y fragment ion series was present in the spectrum, if the signal-to-noise ratios of the fragment ion peaks were only moderate and if relatively intense peaks that could not be assigned to the peptide fragment ion series were present in the spectrum. Spectra were categorized as ‘*poor*’ if only few of the b or y fragment ion series were present in the spectrum and if the signal-to-noise ratios of the fragment ion peaks were low.

### Four-digit HLA typing

Viably frozen PBLs were thawed and used for DNA isolation with the QIAamp DNA Mini kit (Qiagen) according to manufacturer’s instruction. Four-digit HLA imputation was performed at the Human Genomics Facility of the Genetic Laboratory of the Department of Internal Medicine at Erasmus MC. All samples were genotyped on the GSA-MD v3 and data was processed in Genomestudio 2.0. Quality control was performed in PLINK 1.9, using a sample call rate filter of 97.5%^75^. For variants, a Hardy Weinberg Equilibrium threshold of 10Ε-5 and a variant call rate threshold of 90% were applied before using zCall (version May 8^th^, 2012) to improve rare variant calling^76^. Genotype data that passed quality control was imputed in HLA-TAPAS using the multi-ancestry HLA reference panel to obtain 4-digit HLA alleles^77^.

### STRING protein analysis

Source protein annotation was performed in PEAKS Studio 11 and any missing accessions were manually added to the dataset. Protein accessions were filtered for adjuvant-specificity and loaded into the STRING DB webtool available at https://string-db.org/. Protein-protein interaction networks were visualized using a minimal confidence level of 0.7. Adjuvant-specific protein accessions were colored purple if they were found in 3 or more donors, or yellow if found in only 2 donors. The STRING webtool also includes a Gene Ontology (GO) functionality which allows for the identification of upregulated biological processes. Upregulated GO processes were identified from PIC-specific source proteins identified in 2 or more donors using a minimal false discovery rate of 0.05 and a minimal p-value of 0.05 corrected for multiple testing by the Benjamini-Hochberg method.

### Flow cytometric analysis

Cells were plated out into round-bottom 96-well plates (100.000 cells/well; Corning) and washed once with 200 µL block buffer (PBS, 0.1% BSA, 1% human serum (Pan Biotech), 0.02% sodium azide (VWR) before labeling at 4 °C for 30 min in the dark with 50 µL antibody solution in block buffer. Anti-human protein antibodies used for moDC analysis were: CD45-APCeFluor780 (0.31 µg/mL; HI30, Thermo Fisher), CD14-eFluor450 (2 µg/mL; 61D3, Thermo Fisher), CD1a-APC (0.15 µg/mL; HI149, BD Pharmingen), DC-SIGN-PerCP-Cy5.5 (0.08 µg/mL; DCN46, BD Pharmingen), CD11c-FITC (5 µg/mL; KB90, Dako), CD80-FITC (5 µg/mL; MAB104, Thermo Fisher), CD83-APC (0.67 µg/mL; HB15e, Thermo Fisher), HLA-DR-PE (0.02 µg/mL; LN3, Thermo Fisher), CD40-PerCP-eFluor710 (0.08 µg/mL; 5C3, Thermo Fisher), and the viability dye AQUA Live/Dead-AmCyan (1:200; Invitrogen). Anti-human protein antibodies used for primary DC analysis were: CD45-eFluor450 (0.625 µg/mL, HI30, Thermo Fisher), CD19-FITC (2 µg/mL, HIB19, Thermo Fisher), CD3-FITC (1.25 µg/mL, UCHT1, Thermo Fisher), CD14-FITC (2 µg/mL, 61D3, Thermo Fisher), CD56-FITC (0.03 µg/mL, NCAM16.2, BD Biosciences), CD11c-PE-Cy7 (4 µg/mL, 3.9, Thermo Fisher), HLA-DR-PerCP-Cy5.5 (0.06 µg/mL, LN3, Thermo Fisher), BDCA1-APC (1 recommended test volume, AD5-8E7, Miltenyi Biotech), BDCA2-APC (1:10, AC144, Miltenyi Biotech), BDCA3-APC (1.65 µg/mL, AD5-14H12, Miltenyi Biotech), and Fc Block (2.5 µg/mL, BD Pharmingen). Cells were washed with 200 µL block buffer after labeling and measured on the BD FACS Canto (BD Biosciences). Analysis was performed using FlowJo v10.8.1 software (FlowJo).

### T2 HLA binding assay

The T2 cell line (174xCEM.T2, kindly provided by prof. dr. R. Debets) was cultured in RPMI-1640 supplemented with 2 mM ultraglutamin (Lonza), 10% heat-inactivated FCS (PAN biotech) and 100 U/mL penicillin/streptomycin. Based on previously published protocols, T2 cells (0.75 × 10^6^) were incubated with the peptides in serum-free culture medium supplemented with 3 µg/mL β2-microglobulin (Bio-Rad) for 3 h at 37 °C, 5% CO_2_^41,78^. Corresponding concentrations of the established HBV epitope HBcAg_18-27_ and DMSO were taken along as a positive and negative control, respectively. Cells were washed with ice-cold block buffer and labeled with anti-human HLA-A2-AlexaFluor488 (Bio-Rad, 1 µg/mL) for 30 min at 4 °C in the dark. Cells were washed again with ice-cold block buffer prior to flow cytometric analysis on a BD FACSCanto II (BD Biosciences).

### Western blot analysis

To determine the efficiency of HLA-I-IP, PNS samples from the equivalent of 185,000 moDCs were taken post-pre-clear and post-HLA-I-IP. Samples were denatured for 5 min at 95 °C and run on a 10% SDS gel. Proteins were transferred to an Immobilon-FL PVDF membrane (Millipore) and after 1 h block at RT (Licor) probed for HLA-I presence with 0.25 µg/mL anti-human pan-HLA-I antibody (EMR8-5, Abcam) overnight at 4 °C. After extensive washing with PBS-0.05% Tween, the membrane was incubated with 0.1 µg/mL goat-anti-mouse IgG (IRDye 800CW, Licor) for 1 h at RT. Finally, the membrane was washed five times with PBS-0.05% Tween and once with PBS, before measurement with the Odyssey DLx Imaging System (Licor). IP efficiency was calculated by dividing the HLA-I signal in the post-IP sample by the HLA-I signal in the pre-IP signal. For HLA binder correlation analysis, ‘*corrected cellular input’* was determined by correcting the harvest of moDCs for IP efficiency. To estimate *‘HLA input’*, WB-obtained HLA-I signals were normalized against moDC numbers and then multiplied by the *corrected cellular input*.

### Data analysis software, statistics and visualization

The R-script was coded in R studio v4.2.3 (Posit)^79^. Statistical data analysis was performed in Prism v8 (Graphpad); Shapiro-Wilk testing was done to assess data normality. Figures were created (Fig. 1) and compiled in Illustrator 2021 (Adobe).

## Supplementary information

Supplementary data 1

Supplementary information

## Data Availability

All mass spectrometry immunopeptidomics data generated in this study have been deposited to ProteomeXchange Consortium via the PRIDE^74^ partner repository with the dataset identifier PXD051490. Donor informed consent prohibits public sharing of obtained GSA SNP data used for HLA imputation and limits its use to HLA imputation. Please contact the corresponding author for data requests.

## Code availability

The data was processed by an in-house developed R-script which is publicly available on Github under URL https://github.com/wasdoff/HLA-I-peptidome-analysis-of-SLP-fed-human-dendritic-cells-for-therapeutic-vaccine-design.
