# Supplementary material for: HLA I immunopeptidome of synthetic long peptide pulsed human dendritic cells for therapeutic vaccine design
Source: NPJ Vaccines. 2025 Jan 18;10:12. doi: 10.1038/s41541-025-01069-1 (PMC11742953; doi:10.1038/s41541-025-01069-1)
Supplement: Supplementary file 2 — Supplementary information [file 41541_2025_1069_MOESM2_ESM.pdf]

Supplementary information

'HLA I immunopeptidome of synthetic long peptide pulsed human dendritic cells for therapeutic vaccine design'

Supplementary Figures

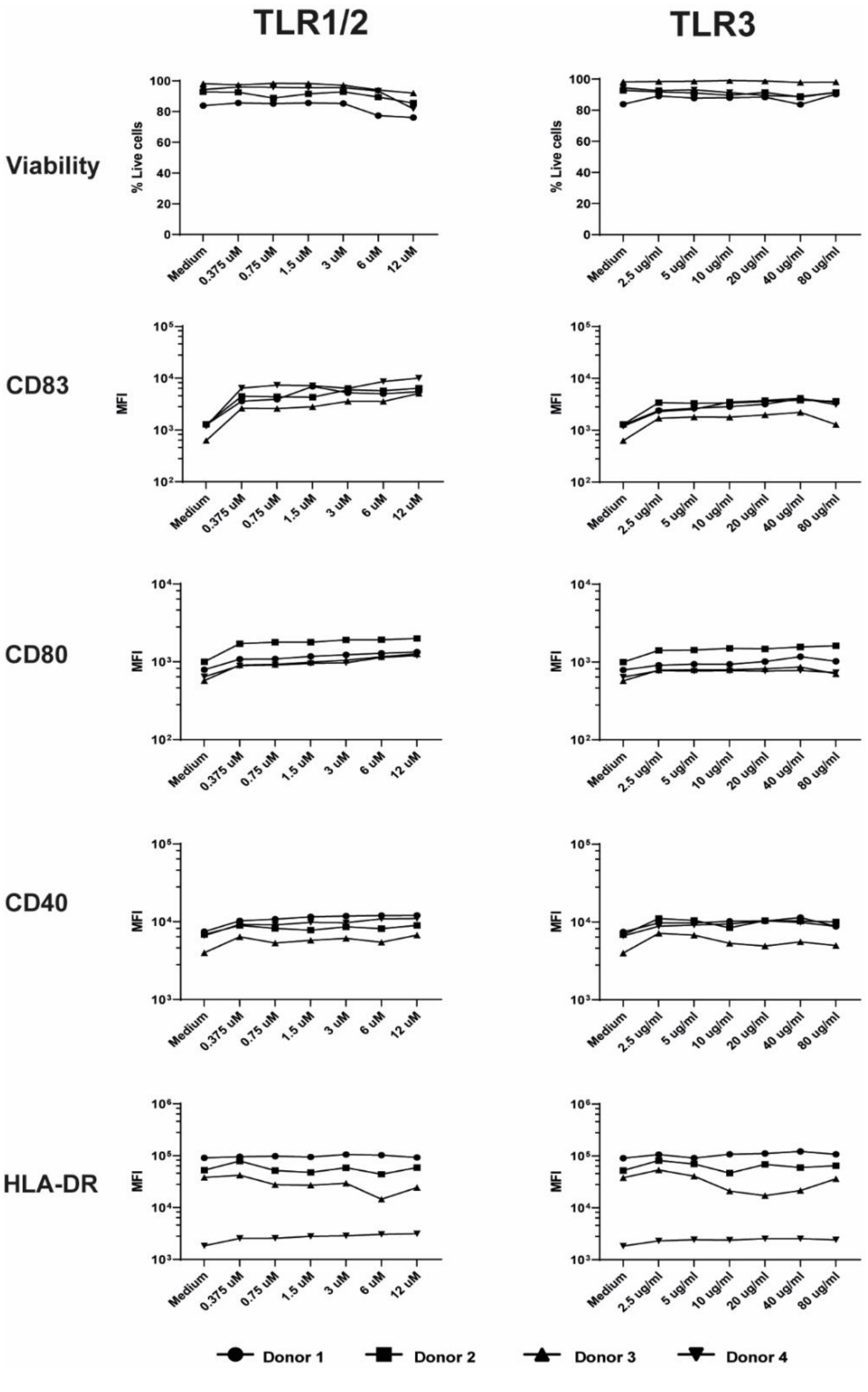

Sup. Fig. 1 | Titration of TLR1/2 ligand Amplivant and TLR3 ligand Poly I:C. Healthy donor moDCs were generated (N=4) and treated for 22 hours with increasing concentrations of either Amplivant (left panels; TLR1/2) or Poly I:C (right panels; TLR3). On day 7 moDCs were harvested and phenotypically characterized by flow cytometric analysis for viability (by viability dye AQUA) and expression of commonly used DC maturation markers. Expression is given in geometric mean values (mean fluorescence intensity, MFI).

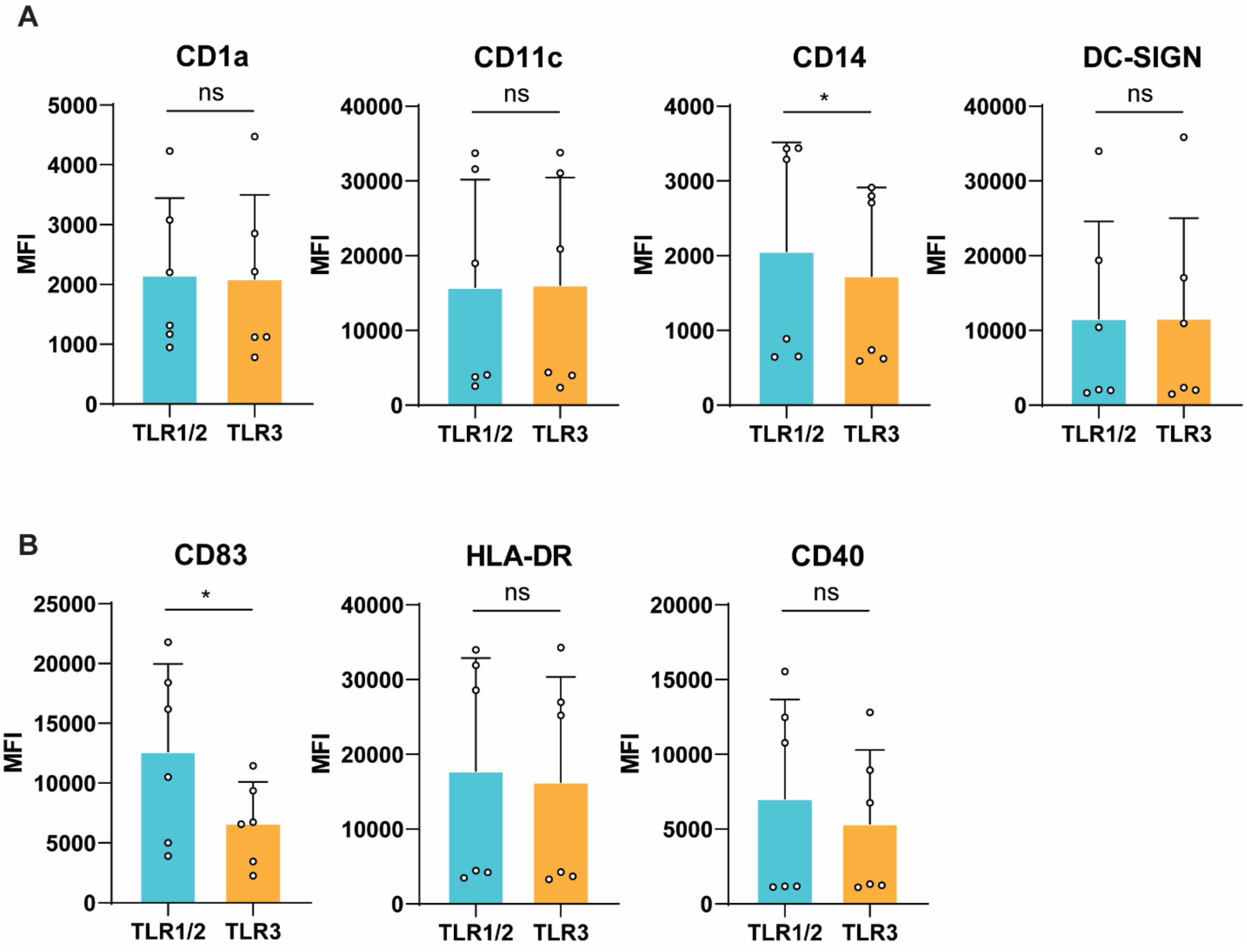

Sup. Fig. 2 | Phenotypical characterization of moDCs upon maturation and pulsing with SLPs. All moDCs were harvested on day 7 and phenotypically characterized by flow cytometric analysis. Mean fluorescent intensity (MFI) was determined for **A.** commonly used DC markers CD1a, CD11c, CD14 and DC-SIGN and **B.** DC maturation markers CD83, HLA-DR and CD40. Bars represent mean ± SD, n=6. Two-tailed paired T-test. Ns = non-significant, * = p<0.05.

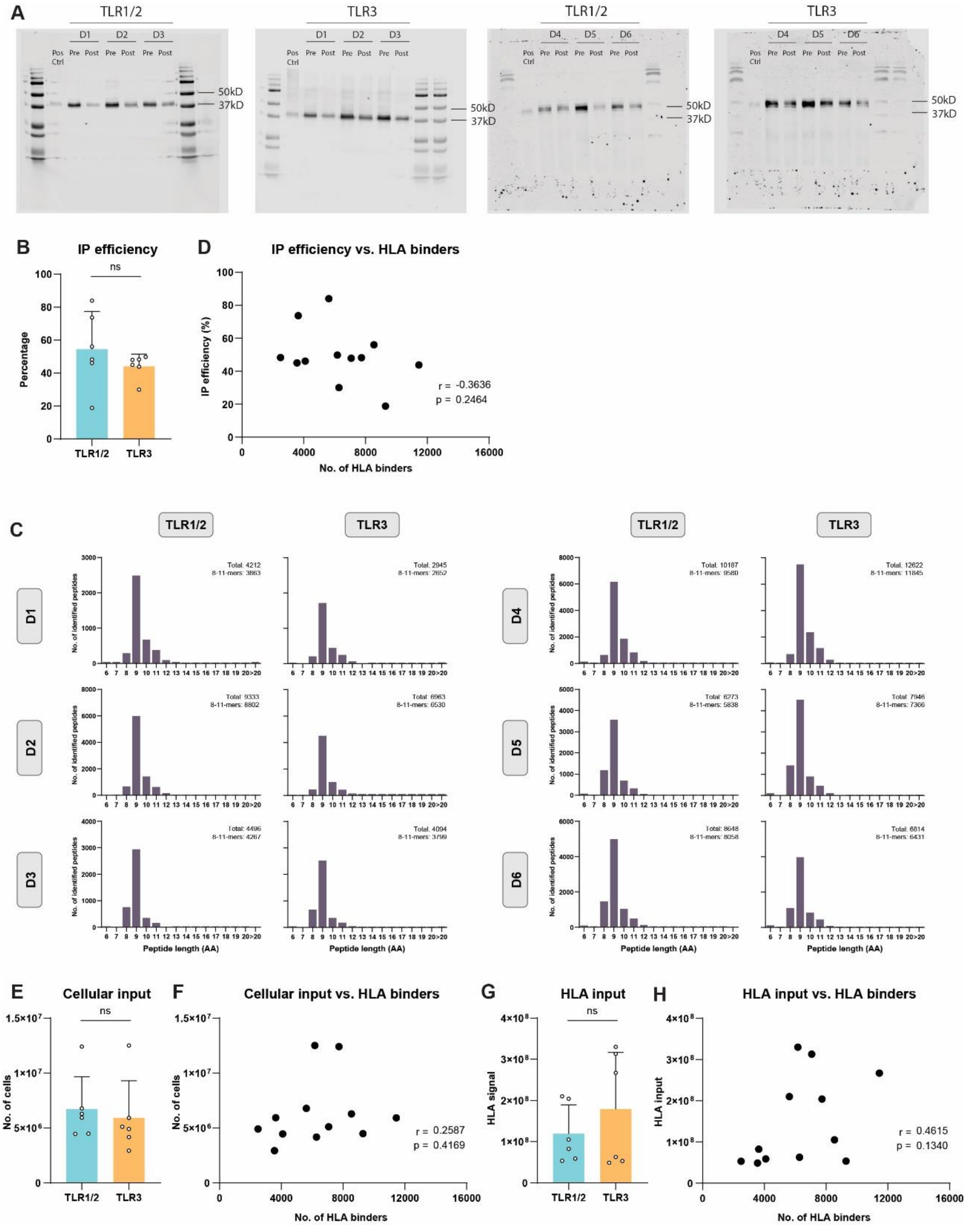

Sup. Fig. 3 | The yield of HLA binders is highly correlated with HLA input. A, B. HLA-I levels in post-nuclear supernatant (PNS) samples were quantified by Western Blot to determine HLA-I IP efficiency. Efficiency was calculated by 100-(Post/Pre*100). "Pos. ctrl"= positive control (PNS 100.000 JY cells), "pre"= pre-IP sample, "post"= post-IP sample. **C.** Peptide length distribution plots for each sample. **D.** Correlation between IP efficiency and the number of predicted HLA binders. **E.** The cellular input per sample used for LC-MS/MS analysis. Determined by correcting total moDC counts for IP efficiency as determined in panel A. **F.** Correlation between cellular input and number of predicted HLA binders. **G.** HLA input per sample for LC-MS/MS analysis. Roughly estimated by multiplying the HLA levels per moDC (blotted HLA-I signal divided by the number of moDCs in PNS) with the cellular input. **B, E, G.** Two-tailed paired T-test. ns = non-significant. **D, F, H.** Two-tailed non-parametric Spearman's rank correlation.

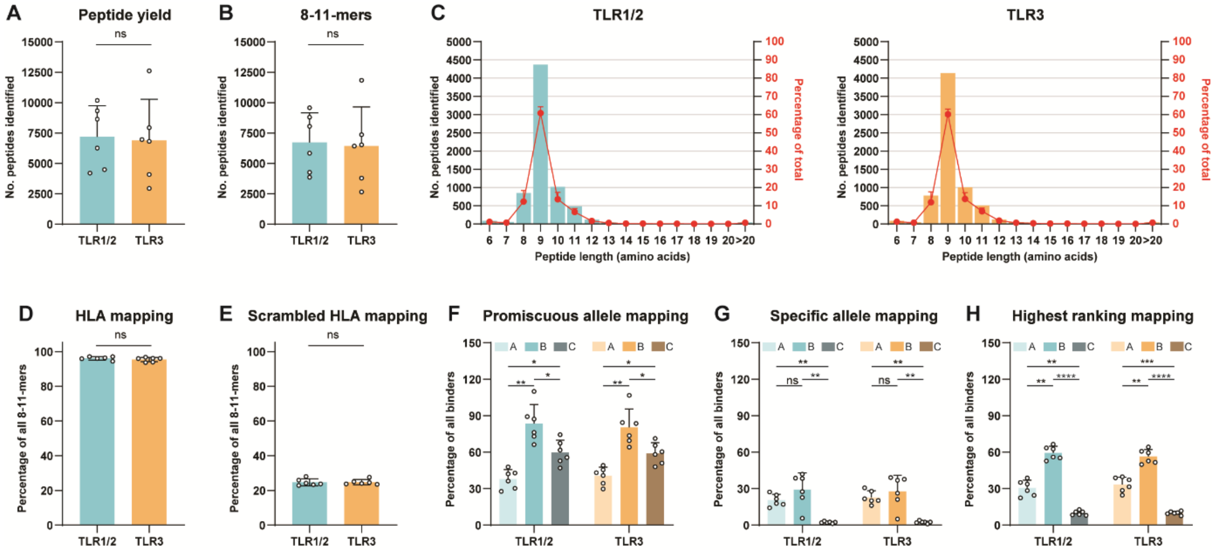

Sup. Fig. 4 | No significant differences in antigen presentation between TLR1/2 and TLR3 stimulated moDCs. moDCs from 6 healthy donors were loaded with 12 HBV SLPs in combination with either a TLR1/2 or TLR3 ligand for 22 hours. HLA-I-peptide complexes were immunoprecipitated and HLA-peptides were identified by LC-MS/MS. The number of **A.** total and **B.** 8-11-mer unique peptides identified. **C.** Length distributions of TLR1/2 samples (blue) and TLR3 samples (orange). Bars represent the mean number of peptides, red symbols represent the percentage of total peptide yield (mean ± SD). **D.** HLA mapping of all 8-11-mers per sample (binding rank ≤2.0%, NetMHCPan4.1) **E.** HLA mapping of scrambled peptide datasets matched per sample in number, length and amino acid composition. **A, B, D, E.** Two-tailed paired T-test. ns = non-significant. **F.** Percentage of all predicted HLA binders mapping to an A-, B-, or C-allele. Predicted binding to an A-, B-, or C- allele does not exclude binding to another allele (*promiscuous mapping*). **G.** Percentage of all predicted HLA-binders mapping to only 1 out of 6 alleles exclusively (*specific allele mapping*). **H.** The percentage of all predicted HLA binders mapping to an A-, B-, or C-allele based on their highest affinity ranking. **F-G.** Each dot represents the cumulative percentage of predicted HLA binders per sample mapping back to the 2 A-, B-, or C-alleles. Repeated measures one-way ANOVA with Geisser-Greenhouse correction, post-hoc Holm-Sidak's multiple comparisons test. ns = non-significant, * = p<0.05, ** = p<0.01, *** = p<0.001, **** = p<0.0001. Plotted for all graphs are means ± SD.

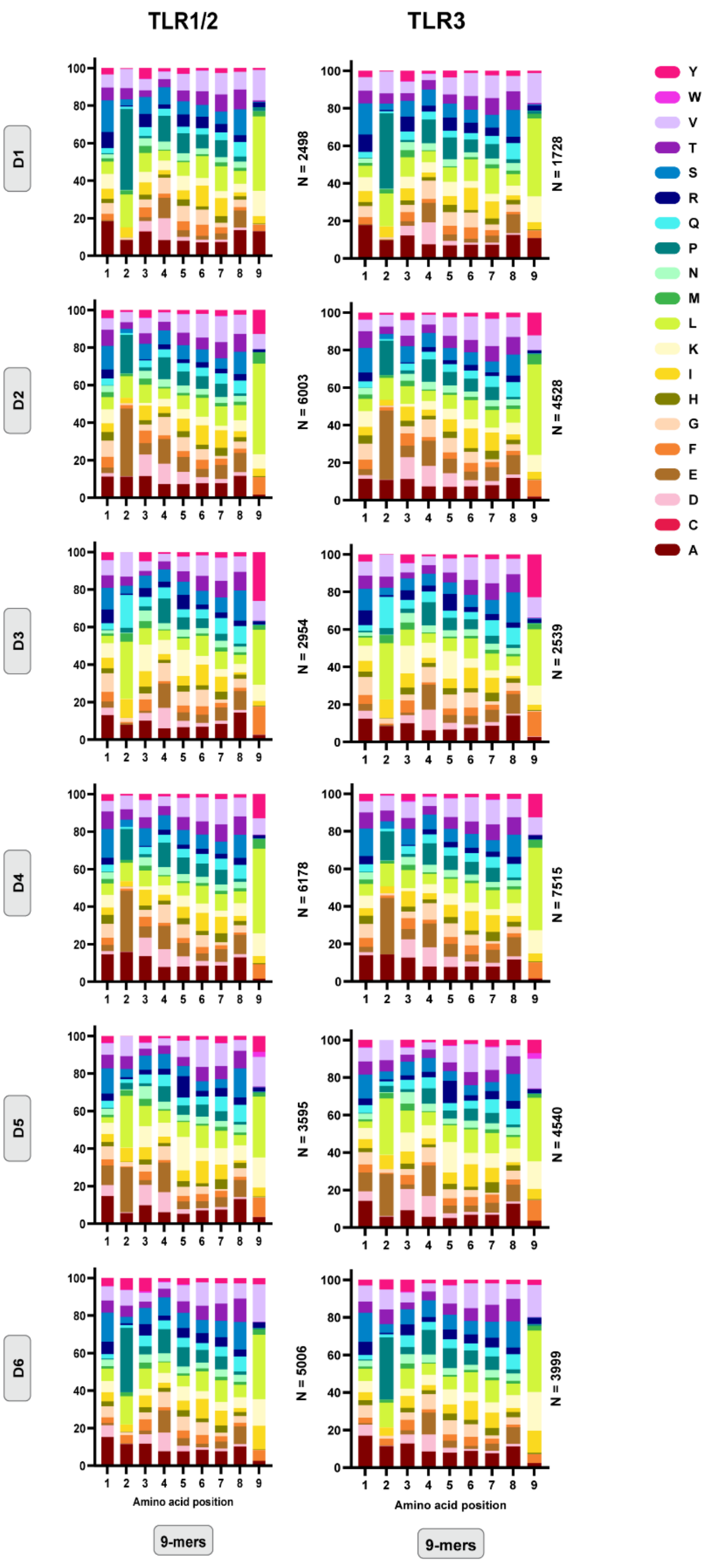

Sup. Fig. 5a | Amino acid distribution of all 9-mers in the TLR1/2 and TLR3 datasets. Each color represents 1 of the 20 amino acids. The number of peptides giving rise to each frequency plot is given vertically on the right of that plot (N=X).

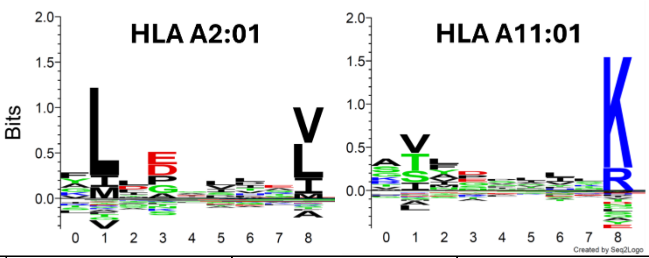

D	AA Freq. /pos	B1 motif	B2 motif	C1 motif	C2 motif
1	1 2 3 4 5 6 7 8 9	07:02	55:01	03:03	07:02
2		35:01	40:01	03:04	04:01
3		08:01	15:01	03:03	07:01
4		35:01	40:01	03:04	04:01
5		08:01	44:02	05:01	07:01
6		07:02	51:01	07:02	14:02

Sup. Fig. 5b | Amino acid distribution of 9-mers eluted from DCs compared to the binding motif for expressed HLA-types. The amino acid (AA) frequency at each position in 9-mer peptides eluted from HLA obtained from DC matured with TLR1/2 ligand Amplivant from all 6 donors were plotted using Seq2Logo and compared against the sequence motifs for the HLA-alleles expressed in each donor obtained from the NetMHCpan-4.1 motif viewer (with integrated Seq2Logo). The AA letter symbols from left to right represent the position in the peptide and the size of the symbol represents the relative frequency of a particular AA at that position. The motifs for the 2 B alleles and 2 C alleles are depicted per donor in the table. The motifs for the common A2:01 and A11:01 alleles are plotted above the Table.

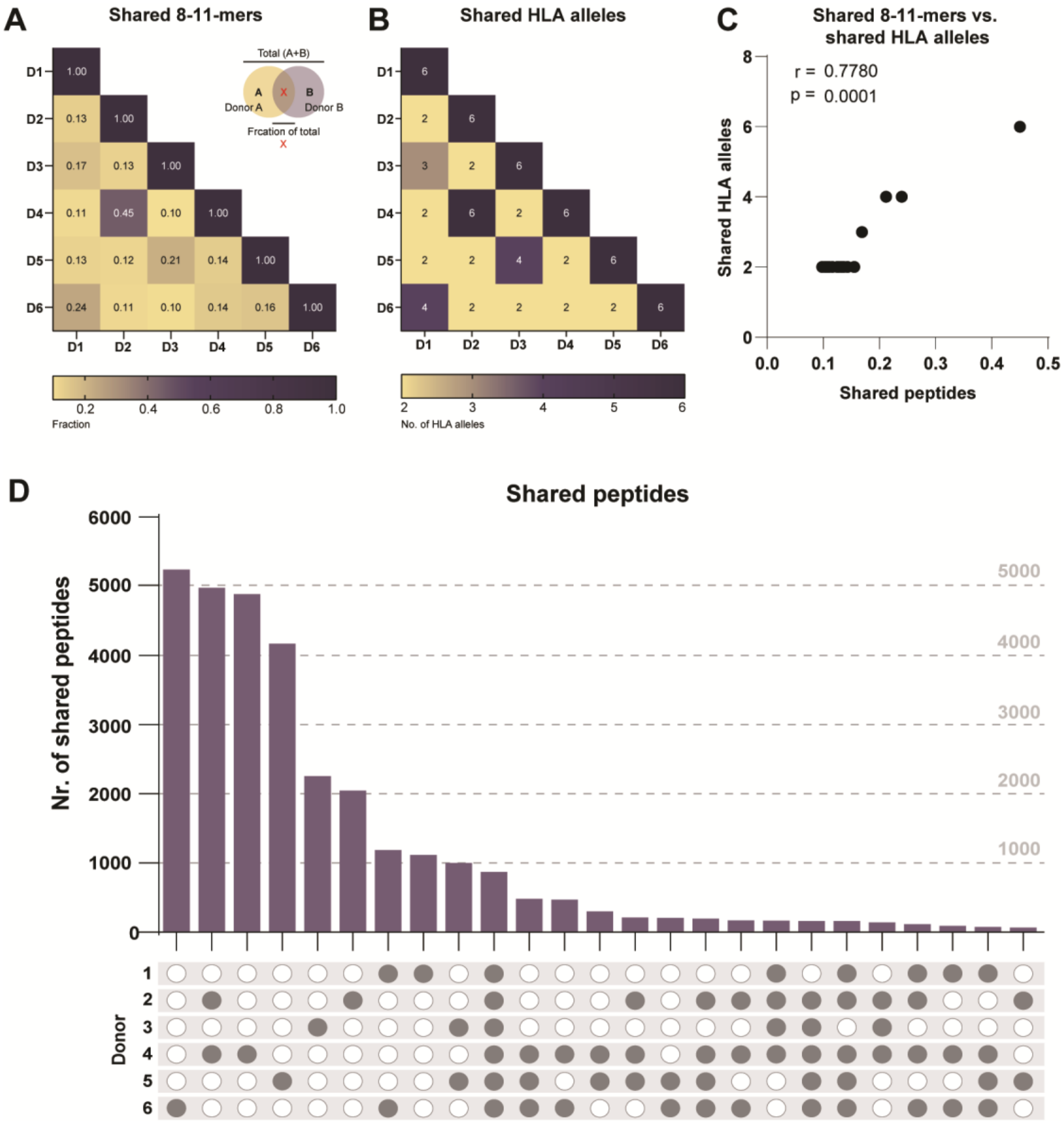

Sup. Fig. 6 | The number of shared 8-11-mers between donors relates to shared B-alleles. A. Correlation matrix of 8-11-mers shared between donors. Overlapping 8-11-mers are given as a fraction (X) of the cumulative number of unique peptide sequences identified in donor A and B. **B.** Correlation matrix of HLA alleles shared between donors (X out of 6). **C.** Correlation plot of the number of shared 8-11-mers and the number of shared HLA alleles. Two-tailed Spearman rank correlation. **D.** Number of (shared) 8-11-mers unique to donors within the dataset. Filled in dots represent peptide identifications within each corresponding donor.

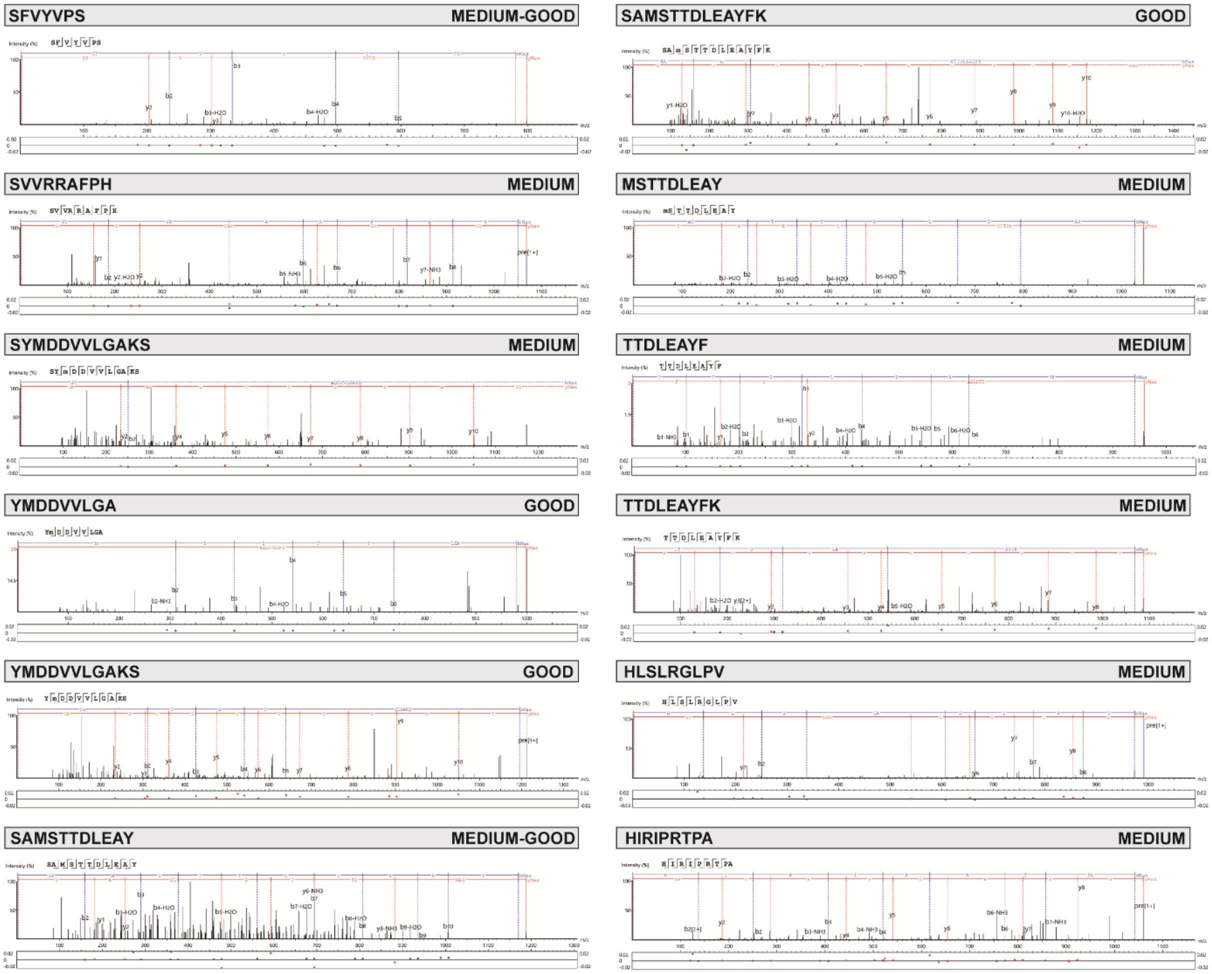

Sup. Fig. 7a | Representative spectra of all SLP-derived peptides. All spectra of SLP-derived peptides were manually checked by an in-house mass spectrometry expert. A representative spectrum was selected of each peptide of sufficient quality.

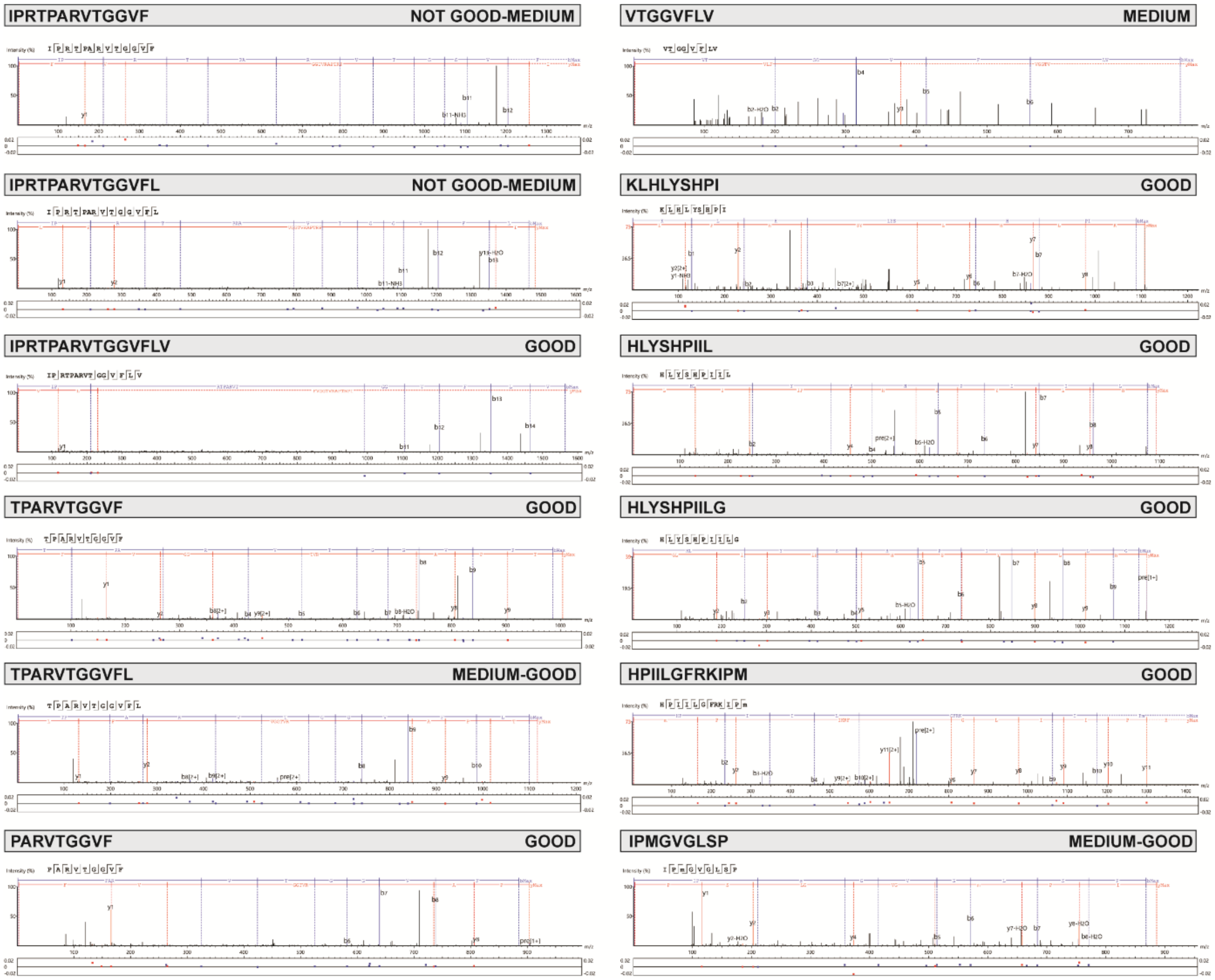

Sup. Fig. 7b | Representative spectra of all SLP-derived peptides. All spectra of SLP-derived peptides were manually checked by an in-house mass spectrometry expert. A representative spectrum was selected of each peptide of sufficient quality.

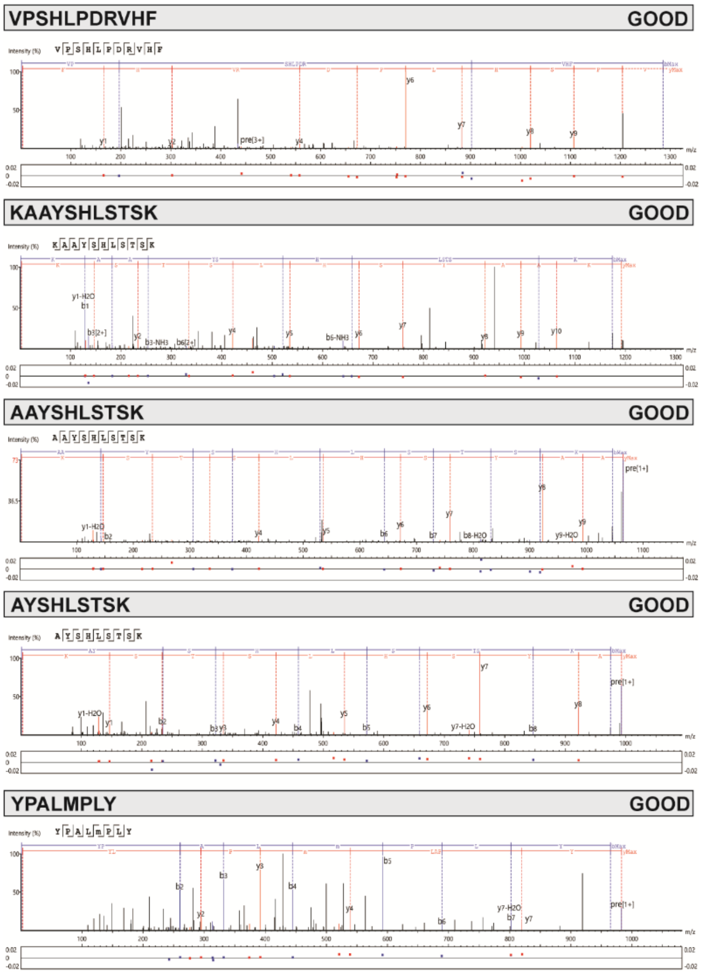

Sup. Fig. 7c | Representative spectra of all SLP-derived peptides. All spectra of SLP-derived peptides were manually checked by an in-house mass spectrometry expert. A representative spectrum was selected of each peptide of sufficient quality.

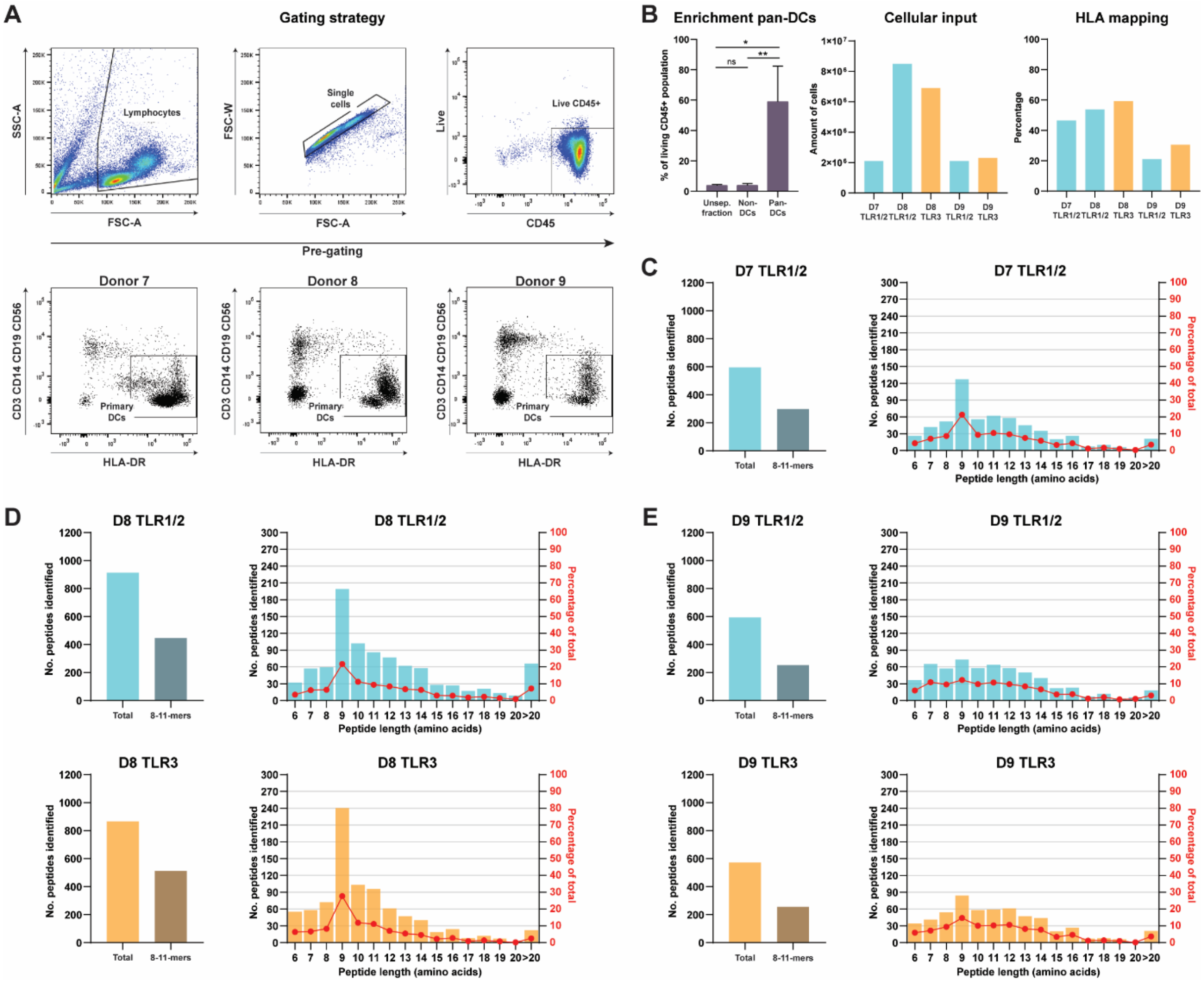

Sup. Fig. 8 | HLA-I immunopeptidomics on primary DCs yields lower amount of HLA peptides. A. Gating strategy for flow cytometric analysis of primary DC enrichment upon MACS sorting. Upper three panels are representative of n=3. **B.** The percentage of primary DCs within the alive CD45+ population in the unseparated fraction (pre-MACS), the positive fraction (non-DCs) and the unlabeled negative fraction (primary DCs) (N=3), as well as the cellular input and HLA mapping in all samples. One-way ANOVA, Friedman's post-hoc multiple comparisons test. ns = non-significant, * = p<0.05, ** = p<0.01. **C-E.** The cellular input (total primary DC count) and length distribution per sample. Note: cellular input not corrected for IP efficiency. Bars represent the mean number of peptides, red symbols represent the percentage of total peptide yield (mean ± SD).

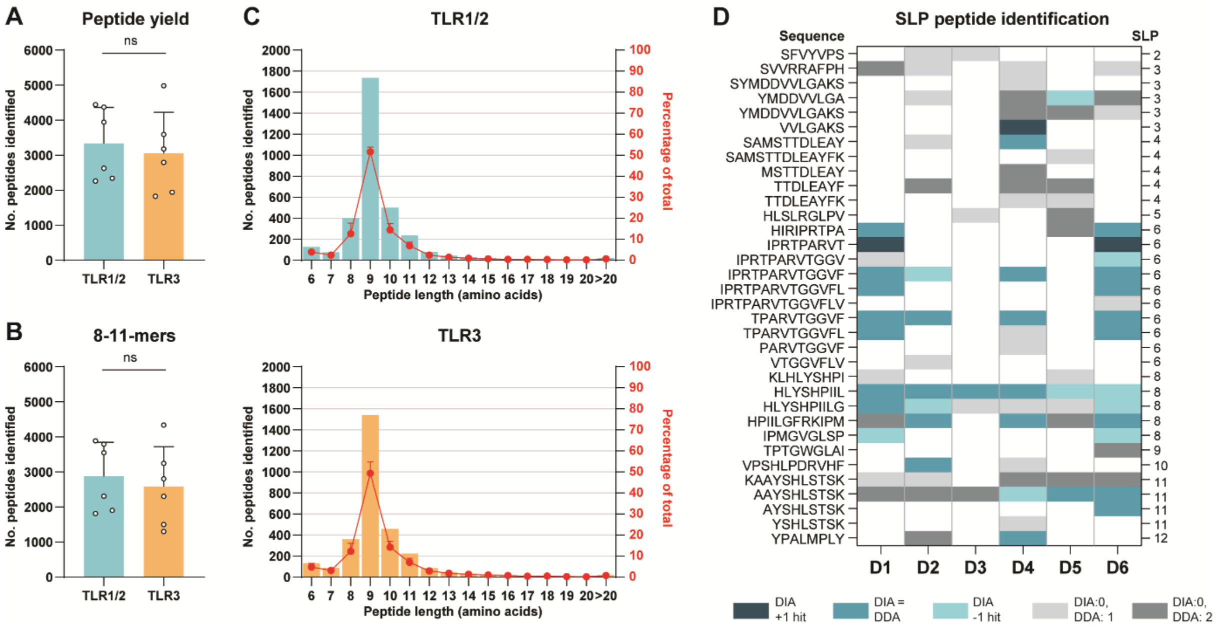

Sup. Fig. 9 | Additional DIA revealed three more SLP identifications. A. The total peptide yield and **B.** number of 8-11-mers identified by DIA analysis. Bars represent mean ± SD. Two-tailed paired T-test. ns = non-significant. **C.** Length distributions of TLR1/2 samples (blue) and TLR3 samples (orange). Bars represent the mean number of peptides, red symbols represent the percentage of total peptide yield (mean ± SD). **D.** Overview of additional SLP peptide identifications by DIA. Given in grey are identifications previously made by DDA, but not reproduced by DIA; in light blue identifications where only 1 out of 2 hits could be reproduced by DIA; in dark blue additional identifications by DIA.

Supplementary tables

SLP sequence	Position	Length
HYFQTRHYLHTLWKAGILYKRETTR	Pol140-164	25
TSFPWLLGCAANWILRGTSFVYVPS	Pol758-782	25
SVVRRAFPHCLAFSYMDDVVLGAKS	Pol535-559	25
LSAMSTTDLEAYFKDCLFKDWEELG	X100-124	25
HLSLRGLPVCAFSSAGPCALRFTSA	X52-76	25
HHIRIPRTPARVTGGVFLVDKNPHN	Pol358-382	25
AARLCCQLDPARDVLCLRPVGAESR	X2-26	25
RKLHLYSHPIILGFRKIPMGVGLSP	Pol499-523	25
ARQRPGLCQVFADATPTGWGLAIGH	Pol688-712	25
SPSVPSHLPDRVHFASPLHVAWRPP	Pol819-843	25
ASSSSSCLHQSAVRKAAYSHLSTSK	Pol269-293	25
GFAAPFTQCGYPALMPLYACIQAKQA	Pol641-666	26

Sup. Table 1 | Overview of synthetic long peptides used in this study. SLPs were designed based on the HBV genome. Twelve SLPs derived from the polymerase and X protein were used in this study ranging between 25-26 amino acids long.

Sample		Cell harvest	IP efficiency	Normalized cellular input	HLA levels (per cell)	HLA input for MS
D1	TLR1/2	8.05E+06	73.7%	5.93E+06	13.9	8.26E+07
	TLR3	10.2E+06	48.3%	4.91E+06	10.9	5.32E+07
D2	TLR1/2	11.2E+06	56.0%	6.28E+06	16.7	10.5E+07
	TLR3	13.9E+06	30.0%	4.17E+06	15.0	6.27E+07
D3	TLR1/2	9.66E+06	46.1%	4.46E+06	13.2	5.89E+07
	TLR3	6.51E+06	45.0%	2.93E+06	16.6	4.87E+07
D4	TLR1/2	23.8E+06	18.8%	4.48E+06	11.9	5.35E+07
	TLR3	13.5E+06	43.8%	5.92E+06	45.1	26.7E+07
D5	TLR1/2	8.08E+06	84.0%	6.78E+06	30.9	21.0E+07
	TLR3	10.7E+06	47.9%	5.11E+06	61.3	31.3E+07
D6	TLR1/2	25.7E+06	48.2%	12.4E+06	16.5	20.4E+07
	TLR3	25.2E+06	49.8%	12.5E+06	26.3	3.30E+07

Sup. Table 2 | HLA-I immunoprecipitation details per sample. For each sample the cell harvest and IP efficiency is stated. The normalized cellular input used for LC-MS/MS analysis is calculated by correcting the cell harvest for the IP efficiency. HLA levels were determined by Western Blot and used to quantify the HLA input for LC-MS/MS analysis by multiplying it by the normalized cellular input.

Term description	Counts	Strength	FDR
Response to virus (GO:0009615)	18/356	0.9	3.18E-07
Defense response to virus (GO:0051607)	15/252	0.98	9.93E-07
Negative regulation of viral genome replication (GO:0045071)	9/56	1.41	1.02E-06
Negative regulation of viral process (GO:0048525)	10/94	1.23	3.17E-06
Regulation of viral life cycle (GO:1903900)	11/139	1.1	6.84E-06
Response to other organism (GO:0051707)	29/1328	0.54	8.16E-06
Response to interferon-alpha (GO:0035455)	6/22	1.64	2.33E-05
Defense response to other organism (GO:0098542)	23/989	0.57	7.50E-05
Immune system process (GO:0002376)	35/2121	0.42	7.82E-05
Immune response (GO:0006955)	26/1321	0.5	2.00E-04
Innate immune response (GO:0045087)	19/754	0.6	3.10E-04
Defense response (GO:0006952)	25/1394	0.45	1.70E-03
Response to interferon-beta (GO:0035456)	5/31	1.41	2.40E-03
Regulation of cytokine-mediated signaling pathway (GO:0001959)	8/152	0.92	5.70E-03
Response to external stimulus (GO:0009605)	33/2355	0.35	5.70E-03
Regulation of response to biotic stimulus (GO:0002831)	11/361	0.68	1.52E-02
Negative regulation of viral life cycle (GO:1903901)	4/27	1.37	2.63E-02
DNA cytosine deamination (GO:0070383)	3/9	1.72	3.16E-02
Regulation of defense response (GO:0031347)	14/638	0.54	3.53E-02
Response to stimulus (GO:0050896)	71/7835	0.16	3.82E-02

Sup. Table 3 | Overview of upregulated biological processes by Gene Ontology analysis of TLR3-specific source proteins. Given from left to right are: term description and GO ID code, source protein counts (identified / contained within category), strength (log-transformed fold increase), and the false discovery rate (FDR).

	A02:01	A11:01	B07:02	B08:01	B15:01	B35:01	B40:01	B44:02	B51:01	B55:01	C03:03	C03:04	C04:01	C05:01	C07:01	C07:02	C14:02
	D1-D6	D1-D6	D1, D6	D3, D5	D3	D2, D4	D2, D4	D5	D6	D1	D1, D3	D2, D4	D2, D4	D5	D3, D5	D1, D6	D6
SVVRRAFPH	35.31	**2.45**	13.95	15.55	7.50	13.29	32.00	31.83	52.57	17.11	18.86	18.86	38.57	45.00	15.11	12.26	12.36
SYMDDVVLGAKS	43.00	**16.48**	75.00	81.43	83.33	75.00	85.00	53.00	80.00	79.23	100.00	100.00	40.50	70.00	45.00	36.67	29.79
YMDDVVLGA	**0.09**	13.74	14.98	7.77	11.46	7.17	10.80	17.58	12.14	3.36	6.53	6.53	0.53	0.40	2.98	3.51	9.68
YMDDVVLGAKS	**4.57**	14.72	57.22	50.61	52.50	55.00	50.00	70.00	63.82	54.38	47.00	47.00	27.35	10.22	60.00	45.00	43.25
SAMSTTDLEAY	36.25	4.48	15.86	41.72	1.26	**0.37**	18.23	8.89	16.51	14.11	8.05	8.05	24.45	20.03	10.19	11.71	9.38
SAMSTTDLEAYFK	72.50	**1.11**	71.25	85.00	50.28	41.33	85.00	47.50	61.47	73.13	37.00	37.00	66.25	42.00	51.67	56.67	52.86
MSTTDLEAY	35.23	6.40	26.19	37.92	1.89	**0.34**	18.19	7.94	16.95	19.31	5.08	5.08	15.57	8.52	4.20	5.68	5.95
TTDLEAYF	29.44	21.05	17.02	17.22	17.87	7.92	16.87	20.52	13.97	54.27	6.11	6.11	0.59	**0.23**	7.01	9.95	9.48
TTDLEAYFK	38.70	**0.30**	53.08	58.79	47.20	21.61	34.67	21.55	48.00	31.64	31.67	31.67	13.60	8.66	14.39	18.78	31.64
HLSLRGLPV	3.63	29.67	4.98	**0.47**	9.39	21.71	29.00	42.00	14.14	5.01	8.76	8.76	11.67	9.84	12.93	14.34	10.00
HIRIPRTPA	18.89	14.97	0.77	2.07	8.69	16.09	37.00	49.50	20.34	**0.24**	9.63	9.63	18.54	29.70	19.53	18.66	14.87
IPRTPARVT	59.55	44.50	0.27	4.56	41.00	4.89	26.25	26.40	2.72	**0.06**	14.94	14.94	10.05	25.41	17.92	21.58	12.00
IPRTPARVTGGV	68.33	85.00	**0.92**	16.49	80.00	31.30	52.50	56.25	5.97	1.53	38.00	38.00	30.86	42.67	49.00	55.00	36.83
IPRTPARVTGGVF	85.00	67.50	**0.47**	10.96	18.54	5.05	38.00	41.00	11.00	5.20	20.46	20.46	19.71	38.67	26.86	28.25	16.20
IPRTPARVTGGVFL	61.88	85.00	**0.55**	9.27	65.00	14.89	41.00	61.67	9.05	5.39	26.60	26.60	18.65	32.67	26.86	21.79	25.13
IPRTPARVTGGVFLV	22.58	85.00	**3.29**	29.24	85.00	40.67	70.00	80.00	8.54	4.26	55.00	55.00	41.00	54.00	62.50	60.00	47.00
TPARVTGGVF	71.25	41.50	**0.19**	6.67	5.33	0.70	27.14	14.69	4.60	2.73	6.82	6.82	12.19	20.94	18.32	22.25	12.92
TPARVTGGVFL	28.89	39.00	**0.58**	9.66	38.90	2.62	12.17	20.43	3.74	3.37	11.29	11.29	8.49	13.66	3.52	5.83	19.02
PARVTGGVF	80.00	67.50	**4.80**	18.53	5.44	8.16	36.33	31.67	12.99	24.67	7.97	7.97	21.45	22.92	22.00	23.86	12.29
VTGGVFLV	10.75	22.67	59.44	47.82	54.44	63.33	60.00	66.67	**8.33**	46.64	21.83	21.83	25.58	9.73	24.44	41.50	36.67
KLHLYSHPI	**1.06**	30.67	10.08	2.97	6.80	32.38	18.77	27.33	16.49	13.33	8.21	8.21	14.06	10.97	12.13	14.03	11.86
HLYSHPIIL	**0.06**	6.31	1.47	0.08	0.64	2.53	2.47	7.53	1.73	1.95	0.21	0.21	0.37	0.68	0.16	0.16	0.53
HLYSHPIILG	**2.98**	29.33	23.56	8.10	14.69	42.33	31.75	49.50	27.05	33.59	15.86	15.86	18.00	19.98	18.27	15.31	22.64
HPIILGFRKIPM	59.55	70.00	**1.49**	3.11	50.00	3.35	53.75	54.00	12.46	4.97	29.67	29.67	39.33	55.00	49.00	51.67	33.89
IPMGVGLSP	36.92	22.61	**0.95**	9.71	29.48	1.31	19.55	26.40	1.23	0.07	14.92	14.92	11.49	21.53	26.00	25.82	18.60
TPTGWGLAI	24.85	28.29	**0.23**	4.31	35.87	0.56	12.88	15.00	0.17	0.33	3.09	3.09	2.50	6.44	8.09	12.21	8.13
VPSHLPDRVHF	31.95	25.60	**0.66**	5.14	11.02	0.71	15.57	8.46	2.21	4.79	10.81	10.81	2.97	10.56	5.05	4.97	8.91
KAAYSHLSTSK	45.33	**0.84**	33.45	68.44	23.82	46.00	62.50	44.00	57.20	36.65	34.00	34.00	30.93	32.67	26.14	20.61	22.06
AAYSHLSTSK	22.86	**0.09**	13.94	33.92	11.05	23.83	23.00	16.86	31.41	6.93	11.74	11.74	18.35	21.50	10.74	12.56	14.51
AYSHLSTSK	32.78	**2.29**	25.20	37.42	19.41	33.88	26.50	15.71	47.80	24.09	32.00	32.00	7.25	39.50	6.76	4.65	3.89
YSHLSTSK	53.67	**3.84**	38.43	37.42	19.26	26.16	52.50	44.00	30.33	14.65	22.67	22.67	18.32	19.52	25.00	27.13	17.42
YPALMPLY	54.67	19.10	3.65	8.95	8.65	**0.08**	20.21	6.50	1.19	2.23	15.55	15.55	2.36	10.95	4.42	2.71	1.93

Sup. Table 4 | HLA mapping of SLP-derived peptides. The HLA mapping of each SLP-derived peptide to all donor HLA alleles was determined with NetMHCpan4.1. A binding rank ≤0.5% is considered a strong binder, 0.5%-≤2% is considered a weak binder. Given in bold and colored are the lowest binding ranks for each peptide (i.e. the highest affinity binding). Green values represent predicted binders, red values represent predicted non-binders.

Supplementary Data

Sup. Data 1 | An overview of all possible isoleucine/leucine (I/L) duplicates in the context of the total dataset of mass spectrometry-identified peptides. All possible I/L duplicates are flagged in this list. The first column “I2L” lists all peptide sequences in the dataset. For simplicity, however, all peptides that were identified to contain either an isoleucine or leucine, are listed in this column as leucine-containing peptides. The second column “Name” lists in which sample the sequence was identified. The third column “Adjuvant” lists whether the peptide was found in a TLR1/2-stimulated DC (“AV”) or in a TLR3-stimulated DC (“PIC”). The fourth column “Sequence” lists all identified peptides that were identified in the sample stated in column “Name” on the same row, and that can be a possible duplicate of each other. E.g., in sample D4 PIC, both sequences AAAAAAAAI and AAAAAAAAL were identified. Identified peptides were not excluded from analysis as to not introduce any bias.
